# Crystal structure and enzymatic properties of chalcone isomerase from the Antarctic vascular plant *Deschampsia antarctica* Desv.

**DOI:** 10.1371/journal.pone.0192415

**Published:** 2018-02-02

**Authors:** Sun-Ha Park, Chang Woo Lee, Sung Mi Cho, Hyoungseok Lee, Hyun Park, Jungeun Lee, Jun Hyuck Lee

**Affiliations:** 1 Unit of Polar Genomics, Korea Polar Research Institute, Incheon, Republic of Korea; 2 Department of Polar Sciences, University of Science and Technology, Incheon, Republic of Korea; Russian Academy of Medical Sciences, RUSSIAN FEDERATION

## Abstract

Chalcone isomerase (CHI) is an important enzyme for flavonoid biosynthesis that catalyzes the intramolecular cyclization of chalcones into (S)-flavanones. CHIs have been classified into two types based on their substrate specificity. Type I CHIs use naringenin chalcone as a substrate and are found in most of plants besides legumes, whereas type II CHIs in leguminous plants can also utilize isoliquiritigenin. In this study, we found that the CHI from the Antarctic plant *Deschampsia antarctica* (*Da*CHI1) is of type I based on sequence homology but can use type II CHI substrates. To clarify the enzymatic mechanism of *Da*CHI1 at the molecular level, the crystal structures of unliganded *Da*CHI1 and isoliquiritigenin-bound *Da*CHI1 were determined at 2.7 and 2.1 Å resolutions, respectively. The structures revealed that isoliquiritigenin binds to the active site of *Da*CHI1 and induces conformational changes. Additionally, the activity assay showed that while *Da*CHI1 exhibits substrate preference for naringenin chalcone, it can also utilize isoliquiritigenin although the catalytic activity was relatively low. Based on these results, we propose that *Da*CHI1 uses various substrates to produce antioxidant flavonoids as an adaptation to oxidative stresses associated with harsh environmental conditions.

## Introduction

**Fig 1:**
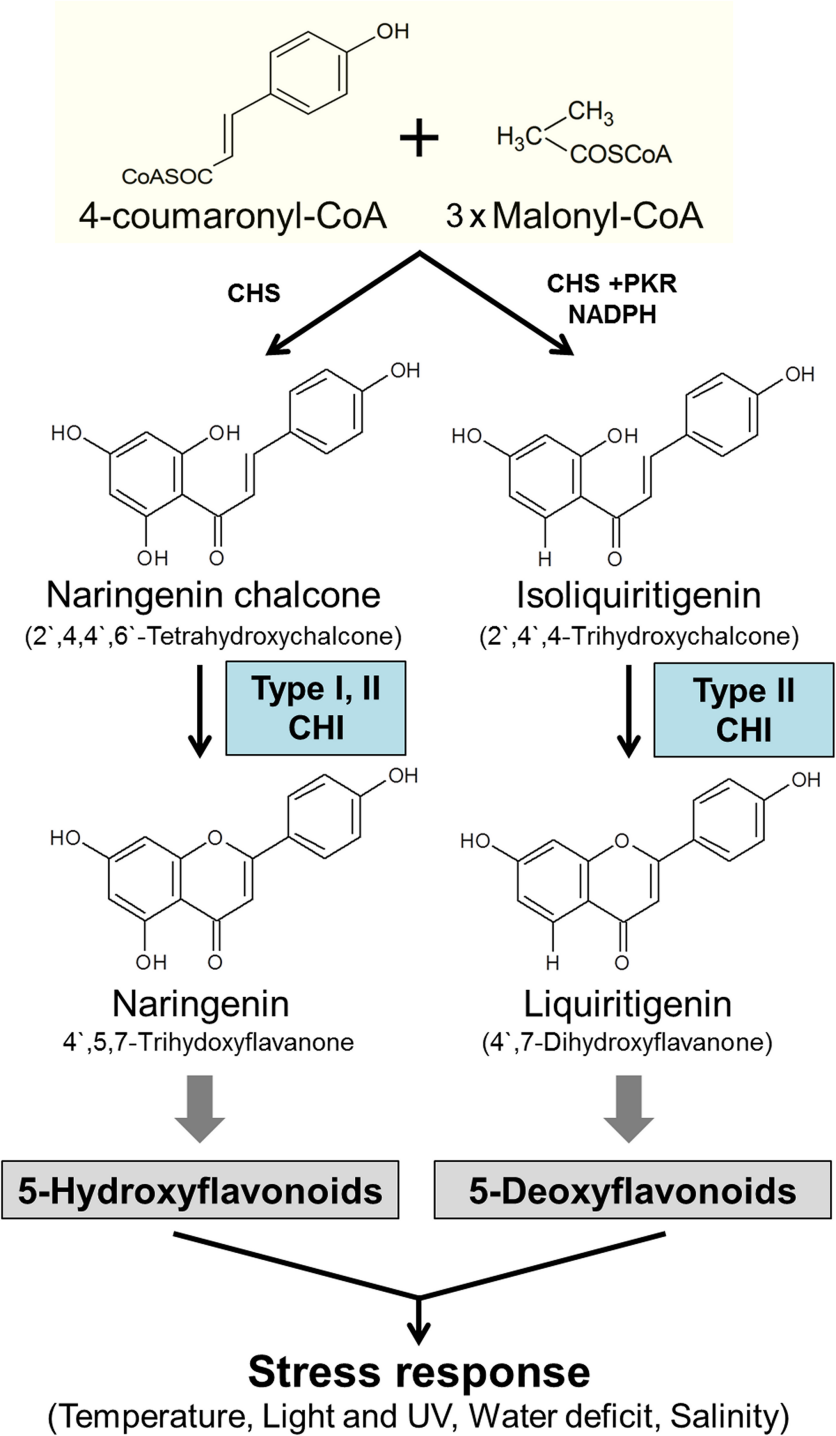
Antioxidant flavonoids produced by *Da*CHI1 in response to various types of oxidative stresses. Substrate structures and specificities of type I and II CHIs are shown. CHS, chalcone synthase; PKR, NADPH-dependent chalcone reductase.

As antioxidant compounds, flavonoids play critical roles in the biological activities of plants, such as protection from ultraviolet (UV) radiation, pathogen resistance, plant coloration, nodulation, auxin transport, and pollen fertility [1–4]. They also have beneficial effects on human health, including the prevention of cardiovascular disease, obesity, and diabetes [5–7]. Thus, genes involved in flavonoid biosynthesis in a wide range of plants are attracting interest for their potential biotechnological applications. In plants, flavonoid biosynthesis begins with phenylpropanoid metabolism in which phenylalanine is transformed into *p*-coumaroyl-coenzyme (Co)A. Chalcone synthase (EC 2.3.1.74), the first enzyme responsible for generating the basic flavonoid skeleton, then catalyzes the condensation of three molecules of malonyl-CoA with one molecule of *p*-coumaroyl-CoA to form chalcone. This is followed by the intramolecular and stereospecific cyclization of chalcone into (2S)-flavanone—a precursor of many downstream flavonoids—by chalcone-flavanone isomerase (CHI; EC 5.5.1.6) (Fig 1). Although the isomerization reaction can proceed spontaneously, the turnover rate is increased 10^7^ fold by CHI-mediated catalysis [8].

To date, a number of CHI genes have been cloned from many different plants including soybean, corn, wheat, alfalfa, peanut, fenugreek, carnation, and *Ginkgo biloba* [8–15]. Flavonoid production can be altered by modulating CHI levels: overexpression of *Glycyrrhiza uralensis* CHI resulted in increased flavonoid production in hairy roots [16], whereas blocking flavonoid biosynthesis by inactivating CHI in onion and tobacco caused chalcone accumulation and reduced flavonoid content [17, 18]. CHIs are classified into two types according to substrate specificity. Type I CHIs—which are found in most plants including barley, rape, petunia, *Arabidopsis*, and rice—isomerize naringenin chalcone (2',4',6',4-tetrahydroxychalcone) into 5-hydroxy-flavonoid; whereas type II CHIs, common in leguminous plants, utilize both naringenin chalcone and isoliquiritigenin (2',4',4-trihydroxychalcone) to synthesize 5-deoxy-flavonoid [19–21]. The substrate specificity of CHIs has been attributed to differences in amino acid sequence; for example, Ser190 and Ile191 in the α6 region of type I CHIs are replaced by Thr and Met, respectively, in type I [22].

The three-dimensional structures of plant CHIs in substrate-free and flavanone-bound forms have been reported, including those from *Medicago sativa* and *Arabidopsis thaliana* [22–24]. CHIs exist as a monomer and the core enzyme structure is characterized by a central antiparallel β-sheet (containing 6–7 β-strands) surrounded by 7–9 α-helices. The structure of a bacterial CHI from the flavonoid-degrading gut bacterium *Eubacterium ramulus* was recently elucidated [25, 26], although it differs significantly from those of plants. Based on structural analyses, the catalytic mechanism of plant CHIs is thought to involve the ionization of a substrate 2'-hydroxyl group and subsequent attack of the α,β-unsaturated double bond of chalcone by reactive 2'-oxyanion during intramolecular cyclization. Extensive hydrogen bonding between the enzyme active site and substrate is important for enzymatic activity [27].

The family of genes encoding CHI in *Deschampsia antartica*—one of the only two vascular plant to naturally colonize the Maritime Antarctic—was recently characterized at the molecular level [28]. The Antarctic is one of the most extreme environments for plants due to low temperatures and water availability as well as high levels of UV-B radiation and salinity, which severely restrict plant growth and development. Three genes encoding CHI have been identified in *D*. *antartica* (*Da*CHIs) whose mRNA expression is differentially induced in response to salinity stress. CHI levels are tightly regulated by environmental stresses such as UV radiation, salinity, and temperature. For example, UV-A irradiation induced transcription of the *CHI* gene in *Brassica rapa* and led to increased production of anthocyanin [29]. Similarly, *CHI* expression was markedly upregulated in response to UV-B and salinity stress in *G*. *biloba* and rice, respectively [15, 30]. Increased CHI gene expression can lead to the accumulation of flavonoids with wide-ranging and potent biological activities that help plants survive in harsh conditions. However, there are limited data available on the stress response and physiological roles of CHI in extremophile plants.

As expected, our enzyme activity assay results indicated that *Da*CHI1 had a strong preference for type I substrates, but it was also able to use a type II substrate (isoliquiritigenin) and exhibited very weak enzymatic activity. The crystal structures of the unliganded and isoliquiritigenin-bound forms of *Da*CHI1 revealed that *Da*CHI1 can bind to type II substrates suggesting that *Da*CHI1 can produce antioxidant flavonoids from various substrates for protection against abiotic oxidative stresses. Although further investigation is needed to explain how type I CHIs discriminate between type I and type II substrates and their degree of substrate selectivity, our results provide useful insights into how plants can survive in an extreme polar environment.

## Material and methods

### Plant material and stress treatments of *D*. *antarctica*

*D*. *antarctica* Desv. (Poaceae) plants were collected close to the King Sejong Station (62°14'29"S; 58°44'18"W) on the Barton Peninsula of King George Island in January 2012 and cultured in tissue culture medium composed of 1× Murashige and Skoog, 2% sucrose, and 0.8% phytoagar (pH 5.7) under a 16:8-h light/dark photoperiod with a light intensity of 30 μmol m^−2^ s^−1^ at 16°C [31].

For cold-stress treatment, plants were transferred to a chamber at 0°C under the same light conditions for 2 days. For UV-B treatment, plants were moved for 1 day to a chamber under UV-B was illuminated by a Philips TL-D/08 15W weathering lamp with wavelength of 290–340 nm (Philips, Amsterdam, The Netherlands) supplemented with white light at 2 kJ m^−2^ day^−1^ after 1 day of dark adaptation. Untreated plants were harvested for each condition as a control. All experiments were performed with two independent biological replicates.

### Reverse transcriptase (RT)- and quantitative real-time (qRT)-PCR analyses

Total RNA was isolated using the RNeasy Plant mini kit (Qiagen, Valencia, CA, USA) with on-column DNase digestion (Qiagen) to remove genomic DNA according to the manufacturer’s instruction. RNA quantity and quality were determined with an ND-1000 spectrophotometer (NanoDrop Technologies, Wilmington, DE, USA) and RNA integrity was confirmed by electrophoresis on a 2% agarose gel. Two independent cDNA synthesis reactions were carried out with 2 μg total RNA using Superscript III First-Strand Synthesis Supermix (Life Technologies, Carlsbad, CA, USA) in 20-μl reaction mixtures containing 4 μl of 1:20 diluted cDNA template, 0.5 μM each primer, and 10 μl Quick Taq HS DyeMix (Toyobo, Osaka, Japan). The amplification protocol was as follows: 95°C for 2 min; 30 cycles of 95°C for 10 s, 58°C for 30 s, and 72°C for 15 s; and 72°C for 5 min. The 18S rRNA gene was used as an internal control. A 5-μl volume of the product was resolved by 1% agarose gel electrophoresis. Sequences of primers used in this study are listed in Table 1.

**Table 1:** Primer used in this study.

Primer	Direction	Sequence (5'→3')
DaCHI_R34A mutant	Forward	CCGGCGCAGGTGTGGCAGGGATGGAGATCG
DaCHI_R34A mutant	Reverse	CGATCTCCATCCCTGCCACACCTGCGCCGG
DaCHI_R34M mutant	Forward	CGGCGCAGGTGTGATGGGGATGGAGATCG
DaCHI_R34M mutant	Reverse	CGATCTCCATCCCCATCACACCTGCGCCG
DaCHI_M36A mutant	Forward	CAGGTGTGCGAGGGGCAGAGATCGGCGGCAAC
DaCHI_M36A mutant	Reverse	GTTGCCGCCGATCTCTGCCCCTCGCACACCTG
DaCHI_M96K mutant	Forward	CACCCAGGTTACTAAGATACTGCCATTGAC
DaCHI_M96K mutant	Reverse	GTCAATGGCAGTATCTTAGTAACCTGGGTG
DaCHI_Q104E mutant	Forward	GCCATTGACCGGTGCAGAATACTCGGAAAAGG
DaCHI_Q104E mutant	Reverse	CCTTTTCCGAGTATTCTGCACCGGTCAATGGC
DaCHI_K108A mutant	Forward	GCAGTACTCCGAGGCAGTGACCGAGAACTG
DaCHI_K108A mutant	Reverse	CAGTTCTCGGTCACTGCCTCGGAGTACTGC
DaCHI_Y116A mutant	Forward	GAAAACTGCGTAGCAGCTTGGAAAGCTGTTGGC
DaCHI_Y116A mutant	Reverse	GCCAACAGCTTTCCAAGCTGCTACGCAGTTTTC
DaCHI_S189A mutant	Forward	GAAGCAGTTCTGGAAGCCATTATTGGAGAAC
DaCHI_S189A mutant	Reverse	GTTCTCCAATAATGGCTTCCAGAACTGCTTC
DaCHI_S189T mutant	Forward	GTGTGAAGCAGTTCTGGAAACCATTATTGGAGAACATG
DaCHI_S189T mutant	Reverse	CATGTTCTCCAATAATGGTTTCCAGAACTGCTTCACAC
DaCHI_I190M mutant	Forward	GAAGCAGTTCTGGAATCCATGATTGGAGAACATGGAGTTAG
DaCHI_I190M mutant	Reverse	CTAACTCCATGTTCTCCAATCATGGATTCCAGAACTGCTTC
DaCHI_rt_F1	Forward	GCCCACTCCCACTTCCTC
DaCHI_rt_R1	Reverse	CTCGCCGATGATGGACTC
Da18sRNA-F	Forward	GGGGGCATTCGTATTTCATA
Da18sRNA-R	Reverse	TTCGCAGTTGTTCGTCTTTC

### *Da*CHI cloning, expression, and purification

The full-length *DaCHI1* gene (Genebank ID: FR714890.1) was synthesized with codon optimization for expression in *Escherichia coli* (Bioneer, Daejeon, Korea) and cloned into the pET-28a vector (Novagen, Madison, WI, USA), which was transformed into *E*. *coli* strain BL21(DE3). The recombinant *Da*CHI1 contained a thrombin cleavage site, N-terminal hexa-histidine tag, and *Nde*I and *Xho*I restriction sites. Site-directed mutagenesis was performed using the primers listed in Table 1. Cells were grown in Luria–Bertani medium containing 50 μg/ml of kanamycin until the optical density at 600 nm was 0.6, and recombinant protein expression was induced overnight with 0.5 mM isopropyl-1-thio-β-d-galctopyranoside at 25°C. Cells were harvested by centrifugation (VS-24SMTi; Vision Scientific, Bucheon, Korea) at 6000 rpm and 4°C for 20 min. The cell pellet was resuspended in lysis buffer composed of 50 mM sodium phosphate, 300 mM NaCl, and 5 mM imidazole (pH 8.0) supplemented with 0.2 mg/ml lysozyme. Cells were disrupted by ultrasonication for 10 min at 35% amplitude on ice, and the lysate was centrifuged at 16,000 rpm for 40 min at 4°C. The supernatant containing *Da*CHI1 was poured into a nickel-nitrilotriacetic acid (Ni-NTA) (Qiagen) pre-packed column. Recombinant *Da*CHI1 bound to the Ni-NTA resin was washed with wash buffer composed of 50 mM sodium phosphate, 300 mM NaCl, 20 mM imidazole (pH 8.0) and eluted with elution buffer composed of 50 mM sodium phosphate, 300 mM NaCl, and 300 mM imidazole (pH 8.0). Eluted *Da*CHI1 was concentrated with an Amicon Ultracel-10 K centrifugal filter (Merck Millipore, Cork, Ireland) and incubated overnight with thrombin at 4°C. The hexa-histidine tag was removed and *Da*CHI1 was purified by gel filtration on a Superdex 200 column (GE Healthcare, Piscataway, NJ, USA) equilibrated with a solution of 20 mM Tris-HCl (pH 8.0) and 150 mM NaCl. Fractions containing *Da*CHI1 were collected and concentrated by ultrafiltration to a concentration of 14.6 mg/ml for crystallization.

### Crystallization and data collection

Initial crystallization screening of *Da*CHI1 and isoliquiritigenin-complexed *Da*CHI1 was carried out in a 96-well sitting drop plate (Emerald Bio, Bainbridge Island, WA, USA) at 293 K. The mosquito crystallization robot (TTP Labtech, Melbourn, UK) was used with several commercially available kits including MCSG I–IV (Micro Lytic, Burlington, MA, USA), SG-1 (Molecular Dimensions, Altamonte Springs, FL, USA), Wizard Classic I–IV (Emerald Bio, Seattle, WA, USA), and SaltRx and Index (Hampton Research, Aliso Viejo, CA, USA). A 200 nl volume of protein solution was mixed with an equal volume of reservoir solution and equilibrated against 80 μl reservoir solution.

Hexagonal-shaped *Da*CHI1 crystals appeared after 2 days of incubation in 0.2 M ammonium sulfate, 0.1 M 2-(N-morpholino)ethanesulfonic acid (MES, pH 6.5), and 30% w/v polyethylene glycol 5000 monomethyl ether (PEG 5000 MME) (SG-1 #16). Initial crystallization conditions of *Da*CHI1 were optimized by varying pH and precipitant concentration. The hanging-drop vapor-diffusion method was carried out in 24-well crystallization plates (Molecular Dimensions) at 293 K. Droplet volume was increased to 1 μl of protein solution and an equal volume of reservoir solution, and was equilibrated against 500 μl reservoir solution. Diffraction-quality crystals were obtained using 0.2 M ammonium sulfate, 0.1 M MES (pH 7.3), and 29% w/v PEG 5000 MME. A single crystal was harvested and soaked in 1 M perfluoropolyether cryo oil for cryo-protection (Hampton Research) under a stream of liquid nitrogen. A 2.7-Å resolution diffraction dataset containing 180 images was collected.

The isoliquiritigenin-complexed S189A mutant of *Da*CHI1 was crystallized in the presence of 2 mM of isoliquiritigenin with 5% dimethylsulfoxide (DMSO). The appropriate stoichiometric ratio of protein to ligand was determined to be 1:5. Small, yellow-colored needle-shaped crystals of isoliquiritigenin-complexed *Da*CHI1 were obtained after 1 day using 0.072 M sodium phosphate monobasic monohydrate and 1.728 M potassium phosphate dibasic (pH 8.2) (SaltRx #70). The initial crystallization conditions were optimized to obtain a mountable single crystal in the same manner as for *Da*CHI1. Optimized hexagonal crystals were obtained using 0.076 M sodium phosphate dibasic and 1.152 M potassium phosphate dibasic (pH 8.2). A crystal was soaked in N-paratone oil (Hampton Research) for cryo-protection, and a diffraction dataset containing 180 images was collected at a resolution of 2.1 Å. Diffraction data for *Da*CHI1 and isoliquiritigenin-complexed *Da*CHI1 were collected at a BL-5C beam line at Pohang Accelerator Laboratory (Pohang, Korea). Datasets were indexed, integrated, and scaled using the HKL-2000 package. Data collection statistics are summarized in Table 2.

**Table 2:** Data collection and refinement statistics.

Dataset	*Da*CHI1	*Da*CHI1 complexed with isoliquiritigenin
X-ray source	PAL 5C beam line	PAL 5C beam line
Space group	*C*222_1_	*P*3_2_21
Wavelength (Å)	0.97933	0.97950
Resolution (Å)	50.00–2.70 (2.75–2.70)	50.00–2.10 (2.14–2.10)
Total reflections	85378	197632
Unique reflections	12542 (621)	22153 (1092)
Average I/σ (I)	39.7 (6.4)	40.4 (3.8)
*R*_merge_^a^	0.085 (0.473)	0.095 (0.585)
Redundancy	6.8 (7.0)	8.9 (8.9)
Completeness (%)^b^	98.3 (99.0)	99.5 (100.0)
**Refinement**		
Resolution range (Å)	32.27–2.70 (2.97–2.70)	41.61–2.10 (2.16–2.10)
No. of reflections in working set	12482	20971
No. of reflections in test set	608 (148)	1154 (55)
No. of amino acid residues	428	232
No. of water molecules	1	218
*R*_cryst_ ^b^	0.222 (0.304)	0.189 (0.241)
*R*_free_^c^	0.278 (0.371)	0.226 (0.284)
R.m.s. bond length (Å)	0.006	0.0200
R.m.s. bond angle (°)	1.141	1.946
Average B value (Å^2^) (protein)	77.88	54.44
Average B value (Å^2^) (solvent)	68.40	61.93

^a^ *R*_merge_ = ∑|<I>—I|/∑<I>.

^b^ *R*_cryst_ = ∑||Fo|—|Fc||/∑|Fo|.

^c^ *R*_free_ was calculated with 5% of all reflections excluded from refinement stages using high-resolution data.

Values in parentheses refer to the highest-resolution shells.

PAL, Pohang Accelerator Laboratory; *R*_cryst_, observed structure factor; *R*_free_, predicted structure factor; R.m.s., root-mean-square.

### Structure determination and refinement

A dataset of unliganded *Da*CHI1 belonging to space group *C*222_1_ was obtained with unit-cell parameters a = 63.769 Å, b = 63.769 Å, and c = 45.407 Å. The Matthews coefficient was 2.39 Å^3^Da^−1^, corresponding to 48.46% solvent [32]. Two molecules were predicted in an asymmetric unit. Molecular replacement was performed for *Da*CHI1 structure determination. A sequence-based National Center for Biotechnology Information Basic Local Alignment Search Tool (NCBI-BLAST) search against the Protein Data Bank (PDB) showed that *Da*CHI1 had high sequence identity (61%) to *A*. *thaliana* (*At*)CHI (PDB code, 4DOI) [24]. A molecular replacement solution was obtained using the MOLREP program of the CCP4 program [33, 34]. The structure model of *Da*CHI1 was manually constructed into an electron density map using the COOT program and refined using the REFMAC5 program [35, 36]. The Phenix program was used for model-building and refinement [37]. The final model showed *R*_cryst_ and *R*_free_ values of 0.222 and 0.278, respectively. Molecular replacement for the isoliquiritigenin-complexed *Da*CHI1 S189A mutant was carried out using unliganded-*Da*CHI1 as the search model. After consecutive rebuilding and refinement, the final model of the isoliquiritigenin-complexed *Da*CHI1 S189A mutant had *R*_cryst_ and *R*_free_ values of 0.189 and 0.226, respectively. The quality of both models was analyzed using MolProbity [38]. The parameters for structure determination and refinement are summarized in Table 2. The final atomic coordinates of *Da*CHI1 and the isoliquiritigenin-complexed S189A mutant *Da*CHI1 have been deposited in PDB under codes 5YX3 and 5YX4, respectively (S1 File).

### Enzymatic activity assay

CHI activity was assayed by spectrophotometry based on the isomerization of naringenin chalcone and isoliquiritigenin at 390 nm. The standard reaction was carried out at 25°C using 50 μM substrate in DMSO, 50 mM potassium phosphate buffer (pH 7.6) containing 1 mg bovine serum albumin, and an appropriate amount of enzyme. The reaction was initiated by adding the enzyme and control reactions were carried out without enzyme. The rate of spontaneous reaction was subtracted from the total rate. The kinetic constants *K*_m_ and *k*_cat_ for both substrates (1–64 μM) were estimated by fitting the data to the Michaelis-Menten equation by nonlinear regression using Prism 5 software (GraphPad, San Diego, CA, USA). The assay was performed with triplicate samples.

### Effect of temperature on activity and stability

The effect of reaction temperature on the activity of *Da*CHI1 was determined under standard conditions at temperatures in the range of 0–70°C. To evaluate the thermal stability of CHI, the enzyme solution was incubated at 0–60°C for 30 min, and residual activity was assayed under standard conditions.

### Determination of optimal pH

To determine the optimal pH of CHI, reactions were carried out at pH values ranging from 3.0 to 10.0 using 50 mM citrate (pH 3.0–6.0), 50 mM potassium phosphate (pH 6.0–8.0), and 50 mM Tris HCl (pH 8.0–10.0) as buffers.

## Results and discussion

### *Da*CHI1 is induced by low temperature and UV radiation

**Fig 2:**
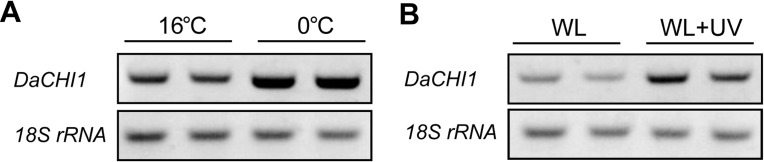
*DaCHI1* transcription under cold and UV irradiation treatments. Plants grown at 16°C and 30 μmol m^−2^ s^−1^ light were subjected to cold and UV-B stress. Plants were exposed to a temperature of 0°C for 2 days or UV-B irradiation (2 kJ m^−2^ day^−1^) for 1 day after 1-day dark adaptation. RNAs extracted from leaves of stress-treated plants were used for RT-PCR of the *DaCHI1* gene, with 18S rRNA as an internal control.

Three *CHI* genes have been reported in *D*. *antarctica* that are differentially expressed at high salinity [28]. A BLAST search of the three genes against the recently reported transcriptome database of *D*. *antarctica* showed that only one assembled contig was a BLAST match, with *DaCHI1* showing the highest identity (100%, 696/696) [39]. We therefore used the *DaCHI1* gene sequence in subsequent experiments. To investigate whether additional abiotic factors affect the transcription of *DaCHI1*, we monitored RNA expression after cold treatment and UV-B irradiation. *DaCHI1* transcription was increased at low temperature and under UV-B treatment (Fig 2). This suggests that *DaCHI1* transcription is regulated by various stressors.

### Overall structure of *Da*CHI1

**Fig 3:**
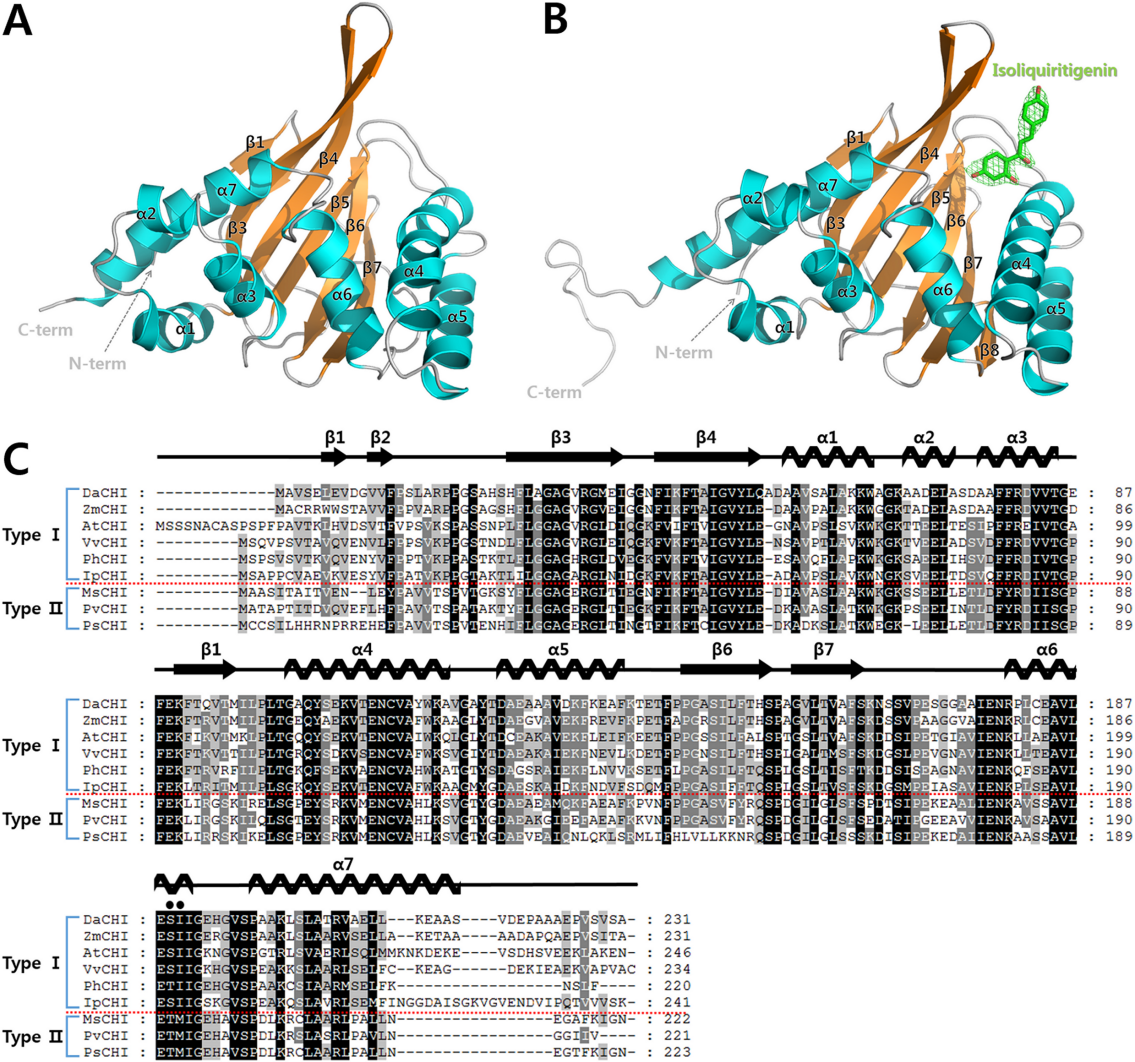
Crystal structure of *Da*CHI1 and multiple sequence alignment of CHI proteins. (A) Overall structure of unliganded *Da*CHI1, shown as a ribbon diagram. α-Helices and β-strands are colored cyan and orange, respectively. (B) Overall structure of isoliquiritigenin-complexed *Da*CHI1, shown as a ribbon diagram. The bound ligand is shown as a stick model with a Fo-Fc electron density map (contoured at 3σ). (C) Multiple sequence alignment of *Da*CHI1, *Zm*CHI (*Zea mays*; UniProtKB code Q08704), *At*CHI (PDB code 4DOI; UniProtKB code P41088), *Vv*CHI (*Vitis vinifera*; NCBI reference sequence NP_001268033.1), *Ph*CHI (*Petunia hybrida*; UniProtKB code P11651), *Ip*CHI (*Ipomoea purpurea*; UniProtKB code O22604), *Ms*CHI (*Medicago sativa*; UniProtKB code P28012), *Pv*CHI (*Phaseolus vulgaris*; NCBI reference sequence XP_007142690.1), and *Ps*CHI (*Pisum sativum*; UniProtKB code P41089). Strictly and partially conserved residues are shaded black and gray, respectively. Two residues for distinguishing between type I and II CHI are indicated by black circles above the sequence alignment. Secondary structures obtained from the crystal structure of *Da*CHI1 are shown above the aligned sequence. Multiple sequence alignment was performed with ClustalX and was edited with the GeneDoc program.

The structures of unliganded *Da*CHI1 and the isoliquiritigenin-complexed catalytically inactive S189A mutant were determined at 2.7 and 2.1 Å resolution, respectively (Table 2). Unliganded *Da*CHI1 was crystallized in the *C*222_1_ space group with two molecules in the asymmetric unit. Our analytical ultracentrifugation results showed that *Da*CHI1 is a monomer in solution with a standardized sedimentation coefficient of 2.14 (S1 Fig). The final model of the unliganded *Da*CHI1 structure contained 218 amino acid residues in a β-sandwich fold with seven α-helices (α1–α7) and seven β-strands (β1–β7). The β1 and β2 strands formed a short β-hairpin structure and β3–β7 strands formed an antiparallel β sheet. The seven α-helices were located at the concave surface of β-sheets and the active site was formed between the antiparallel β-sheet and α-helices (Fig 3A). A structural similarity search with the DALI server showed that *At*CHI (Z score = 32.9) and CHI from *M*. *sativa* (*Ms*CHI; PDB code 1JX1; Z score = 29.1) had high hit scores (Table 3) [23, 24, 40], sharing 61% and 50% amino acid sequence identity, respectively, with these two proteins.

**Table 3:** Structural homologs of *Da*CHI1 selected from a DALI search (DALI-lite server).

Protein	PDB code	DALI score	Sequence % ID with *Da*CHI1 (aligned/total number of residues)	Ligand	Reference
*At*CHI	4DOI	32.9	59% (212/213)	−	[24]
*Ms*CHI	1JX1	29.1	49% (209/218)	5-Deoxyflavonone	[23]
*Ms*CHI	1EYQ	29.1	50% (209/212)	Naringenin	[22]
*Ms*CHI	1JEP	29.0	50% (209/212)	4'-Hydroxyflavonone	[27]
*Ms*CHI	1FM7	29.0	50% (209/212)	5-Deoxyflavonone	[27]
*At*CHIL (At5g05270)	4DOK	26.2	28% (203/208)	−	[24]
AtFAP3 (At1g53520)	4DOL	25.2	25% (203/207)	Palmitic acid	[24]
AtFAP1 (At3g63170)	4DOO	21.1	22% (192/203)	Lauric acid	[24]
Cytochrome P460	2JE2	4.8	11% (104/157)	−	[41]

**Fig 4:**
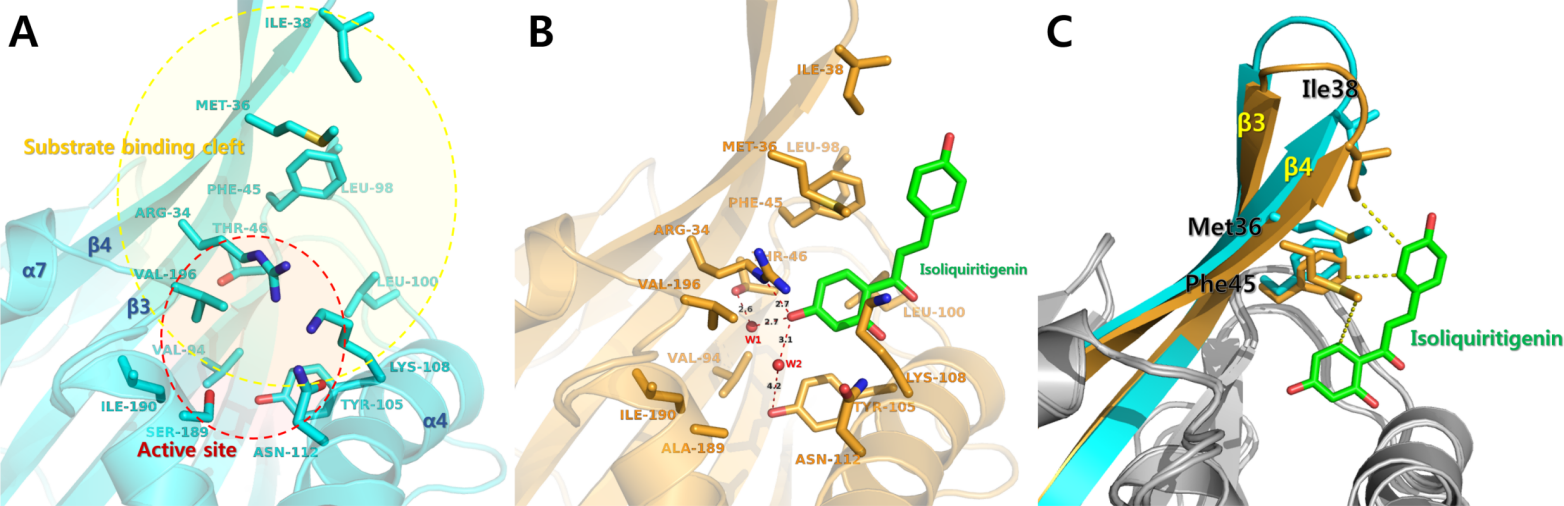
*Da*CHI1 ligand-binding site. (A) Empty ligand-binding site and (B) active site residues of the unliganded *Da*CHI1 structure. The isoliquiritigenin binding mode of *Da*CHI1 is shown. The ligand is depicted as a stick figure with green carbon atoms; interacting residues of *Da*CHI1 are shown as bright orange sticks. Hydrogen bonds with bound isoliquiritigenin are indicated by a red dotted line. (C) Structural superposition of the ligand-binding regions of unliganded *Da*CHI1 (cyan) and isoliquiritigenin (green)-bound *Da*CHI1 (bright orange) showing the closing of the β3–β4 region by ligand binding. Hydrophobic interactions are indicated by yellow dotted lines.

Although *Da*CHI1 was classified as a type I CHI based on multiple sequence alignment, the results of the activity assay showed that *Da*CHI1 utilized type II-specific substrates, although it demonstrated a 1000:1 preference for type I over type II substrates [28]. The wild-type *Da*CHI1 *k*_cat_ values for naringenin chalcone and isoliquiritigenin were 7819 ± 825 min^–1^ and 1.3 ± 0.1 min^–1^, respectively (Table 4). We therefore resolved the crystal structure of isoliquiritigenin-bound *Da*CHI1 to analyze its type II substrate binding mode. Isoliquiritigenin-complexed *Da*CHI1 was crystallized in the *P*3_2_21 space group with a single molecule in the asymmetric unit. The final model had refined *R*_cryst_ and *R*_free_ values of 0.189 and 0.226, respectively. An electron density map of isoliquiritigenin was observed near the entrance to the substrate-binding cleft (Fig 3B). Isoliquiritigenin was bound via hydrophobic interactions with Met36, Ile38, Phe45, and Leu100. It was previously demonstrated that three water molecules are present in the active site of *Ms*CHI that form a hydrogen bond network [23, 27]. In particular, a catalytic water molecule activated by Tyr106 (Tyr105 in *Da*CHI1) functions as a general acid that stabilizes reaction intermediates. Notably, the 4'-hydroxyl group of isoliquiritigenin interacted directly with the NE atom of Arg34. The 4'-hydroxyl group of isoliquiritigenin also formed hydrogen bonds with Thr46 and Tyr105 mediated by two water molecules each (Fig 4B). A structural alignment of the Cα atoms of *Da*CHI1 complexed with isoliquiritigenin yielded a root-mean-square deviation value of 0.437 Å over 167 residues, indicating a similar overall structure. However, ligand binding caused an open/close movement around the strands of the β3–β4 region. As isoliquiritigenin was bound between β3 and the α4 helix, it interacted with Met36 and Ile38 residues in β3 and Phe46 in β4, creating a hydrophobic cleft (Fig 4C). Notably, the structure of the *Da*CHI1-isoliquiritigenin complex revealed how *Da*CHI1 can utilize a type II substrate with moderate selectivity. The substrate-binding cleft of *Da*CHI1 was conserved, with hydrophobic residues from β3 (Met36 and Ile38), β4 (Ile43, Phe45, and Ile48), β5 (Val94 and Met96), Leu100 of loop 97–101, α4 (Tyr105 and Val109), α6 (Ile190), and Val196 of loop 192–197. Several important catalytic residues including Arg34 (Arg36), Thr46 (Thr48), Tyr105 (Tyr106), Lys108 (Lys109), Asn112 (Asn113), and Ser189 (Thr190) were also preserved (residues in parentheses refer to those in *Ms*CHI) (Fig 4A).

**Table 4:** Steady-state kinetic parameters of wild-type and mutant *Da*CHI1.

	Naringenin chalcone			Isoliquiritigenin		
	*k*_cat_ (min^–1^)	*K*_m_ (μM)	*k*_cat_/*K*_m_ (M^–1^ s^–1^)	*k*_cat_ (min^–1^)	*K*_m_ (μM)	*k*_cat_/*K*_m_ (M^–1^ s^–1^)
*Da*CHI1	7819 ± 825	8.5 ± 3.1	1.53 × 10^7^	1.3 ± 0.1	15.3 ± 1.5	1416
R34A	–	–	–	0.1 ± 0.03	5.3 ± 7.8	314
R34M	–	–	–	0.1 ± 0.08	24.3 ± 28.2	69
M36A	2112 ± 239	4.8 ± 2.0	7.33 × 10^6^	0.5 ± 0.15	16.4 ± 10.1	508
M96K	468 ± 51	10.5 ± 3.4	7.43 × 10^5^	–	–	–
Q104E	8082 ± 267	6.1 ± 0.7	2.208 × 10^7^	1.4 ± 0.06	22.5 ± 2.3	1011
K108A	3416 ± 234	4.8 ± 1.2	1.19 × 10^7^	0.2 ± 0.11	11.2 ± 15.9	298
Y116A	578 ± 68	7.0 ± 2.8	1.38 × 10^6^	0.4 ± 0.04	27.6 ± 5.4	242
S189A	299 ± 15	2.4 ± 0.6	2.08 × 10^6^	0.2 ± 0.03	18.5 ± 6.9	180
S189T	13690 ± 556	9.9 ± 1.2	2.306 × 10^7^	1.0 ± 0.04	10.8 ± 1.2	1543
I190M	11860 ± 539	8.6 ± 1.2	2.292 × 10^7^	0.3 ± 0.03	8.0 ± 2.6	625

*K*_m_ and *k*_cat_ were calculated using the Michaelis–Menten equation. Reactions were performed in potassium phosphate buffer (pH 7.6) as described in Material and Methods. All *K*_m_ and *k*_cat_ values are expressed as the mean ± standard deviation of three parallel experiments.

### Structural comparison between *Da*CHI1 and *Ms*CHI

**Fig 5:**
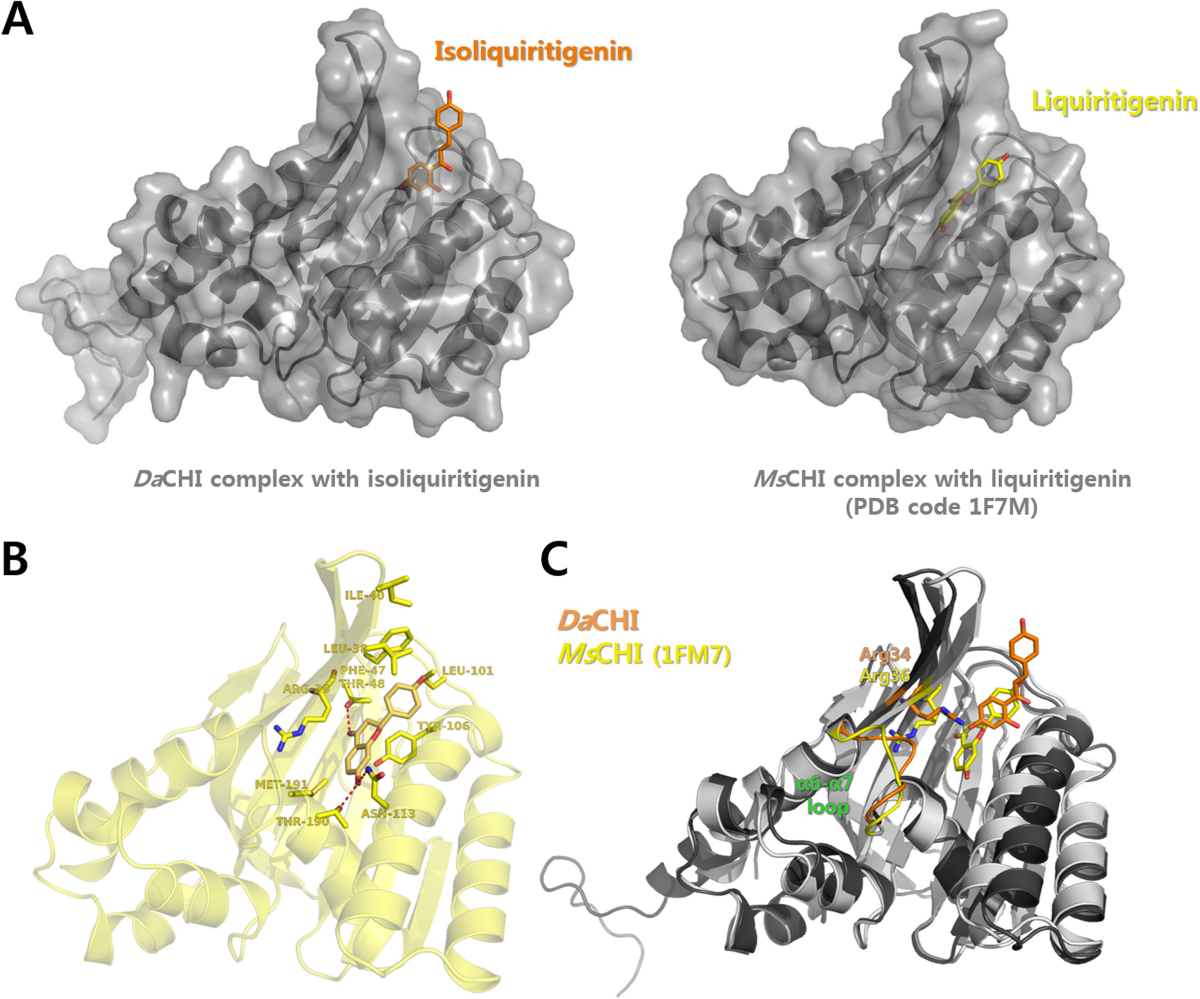
Structural comparison between isoliquiritigenin-bound *Da*CHI1 and liquiritigenin-bound *Ms*CHI. (A) Isoliquiritigenin (orange)-bound *Da*CHI1 and liquiritigenin (yellow)-bound *Ms*CHI structures are shown. (B) Liquiritigenin-binding mode of *Ms*CHI. (C) Superposition of isoliquiritigenin (orange)-bound *Da*CHI1 (dark gray) and liquiritigenin (yellow)-bound *Ms*CHI (light gray) structures. Overall ligand-binding modes differed markedly between the two complexes; the liquiritigenin-binding position was shifted deep into the active site cavity in *Ms*CHI as compared to isoliquiritigenin binding in *Da*CHI1. Conformational differences between the two structures were also observed at the Arg34 position (Arg36 in *Ms*CHI) and in the β6–β7 loop region.

Structural superimposition of isoliquiritigenin-complexed *Da*CHI1 with liquiritigenin-complexed *Ms*CHI (PDB code 1FM7) revealed structural changes induced by ligand binding [27]. Liquiritigenin is a flavanone that is generated from isoliquiritigenin by CHI. In the liquiritigenin-complexed *Ms*CHI structure, liquiritigenin was found to bind deep within the active site and directly interact with thee catalytic residues Thr48, Asn113, and Thr190. However, isoliquiritigenin, which is a chalcone, exhibited a distinct mode of interaction, binding to the putative entrance of the hydrophobic binding pocket of *Da*CHI1 (Fig 5A and 5B).

In the isoliquiritigenin-complexed *Da*CHI1 structure, the 4'-hydroxyl group of isoliquiritigenin interacted with the NE atom of Arg34. However, in the flavanone-complexed MsCHI structure, Arg34 (Arg36 in *Ms*CHI) did not interact with the ligand; it was instead rotated to the opposite side and interacted with Asp200 (Asp202 in *Ms*CHI) located in α7. This alternate conformation of Arg34 repositioned the α6–α7 loop near the active site (Fig 5C). This suggests that Arg34 plays an important role in the catalytic activity of CHI. The R34M and R34A mutants showed significantly reduced activity in the biochemical assay (Table 4). Moreover, the conformational change in the α6–α7 loop may be critical for ligand repositioning since this movement may allow the ligand to bind deep within the active site and interact with the catalytic residues. Collectively, this structural evidence may explain ligand movement and changes in their interaction with CHI. However, it should be noted that the S189A catalytic mutant protein was used to resolve the crystal structure of the *Da*CHI1-isoliquiritigenin complex; therefore, the isoliquiritigenin molecule could not go deep inside the substrate-binding pocket. The bound isoliquiritigenin also made contact with two neighboring protein molecules. As such, we cannot rule out the possibility that crystal packing inhibits the deep binding of isoliquiritigenin in the *Da*CHI1 active site.

### Effect of temperature and pH on *Da*CHI1 activity

**Fig 6:**
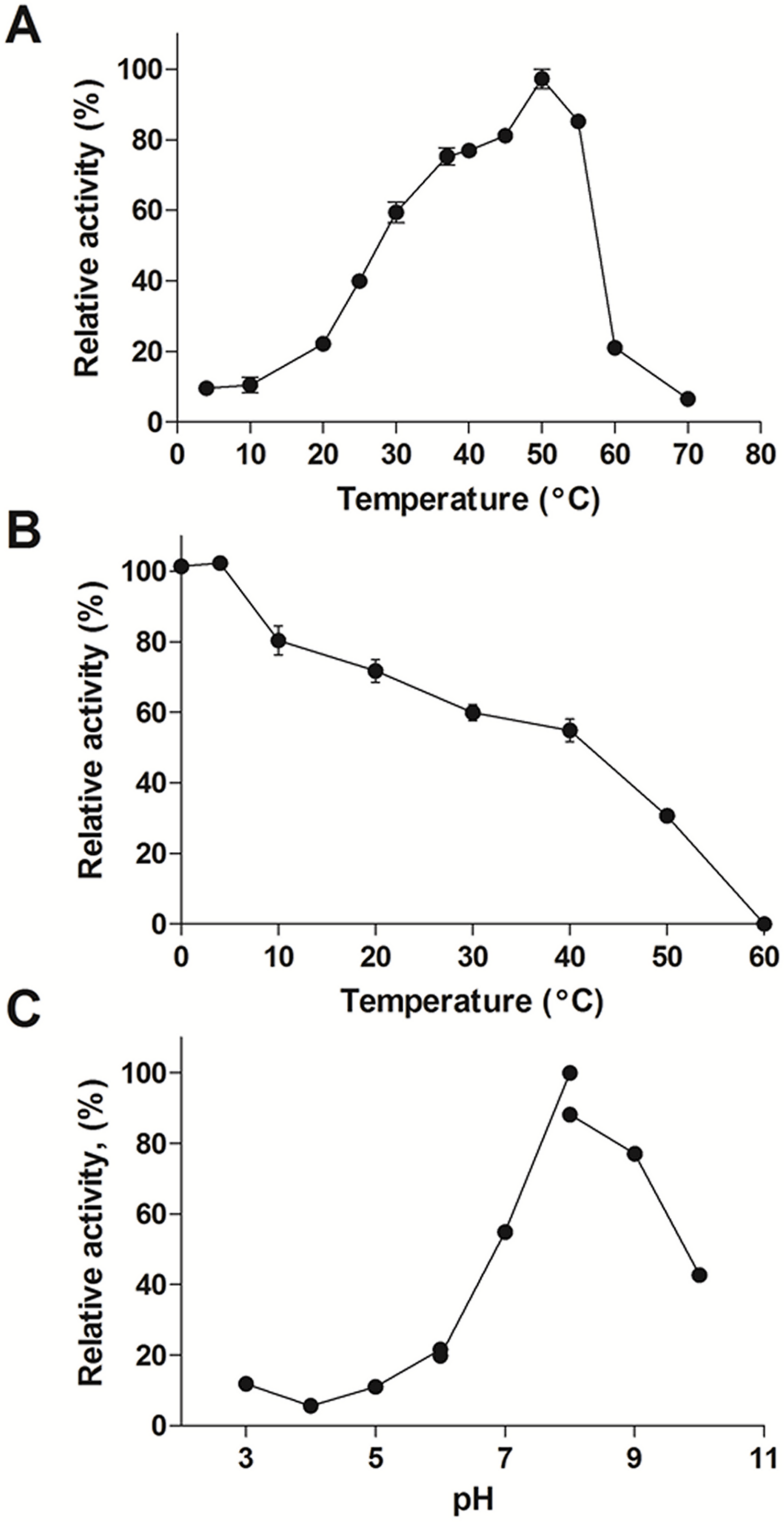
Activity and stability of *Da*CHI1. (A) Effects of temperature on *Da*CHI1 activity. Enzymatic activity was evaluated in the temperature range of 0–70°C in 50 mM potassium phosphate buffer (pH 7.6). Activity is expressed as a percentage of the maximum activity (100%). (B) Effects of temperature on the stability of *Da*CHI1. The enzyme was pre-incubated for 30 min at temperatures ranging from 0–60°C. Residual activity was measured at 25°C. (C) pH dependence of *Da*CHI1 activity. The reaction was carried out at 25°C in buffers with pH ranging from 3.0 to 10.0. The following buffers were used: pH 3.0–6.0, 50 mM citrate; pH 6.0–8.0, 50 mM potassium phosphate; and pH 8.0–10.0, 50 mM Tris-HCl. All measurements were made in triplicate.

Since *Da*CHI1 is derived from *D*. *antarctica*, a plant that is highly tolerant to freezing, we examined the effects of temperature on enzymatic activity in the temperature range of 0–70°C with isoliquiritigenin as a substrate. The optimum temperature for recombinant *Da*CHI1 activity was 50°C (Fig 6A). The enzymatic activity rapidly decreased at temperatures over 60°C, and was almost completely lost at 70°C. In contrast to many known cold-active enzymes that show > 80% of its maximum activity at temperatures below 10°C, the activity of *Da*CHI1 was 22% of the maximum value at 20°C. Moreover, *Da*CHI1 had a higher optimum temperature than its mesophilic counterparts in *G*. *biloba* and the anaerobic bacterium *E*. *ramulus* (30°C and 45°C, respectively) [15, 26]. We also investigated the effect of temperature and pH on the stability of *Da*CHI1. The enzyme showed greater than 50% activity after 30 min of pre-incubation at 40°C, whereas the activity was completely lost after pre-incubation at 60°C (Fig 6B). Moreover, activity was highest at neutral pH (around 8.0; Fig 6C), consistent with the pH optimum of known CHIs (pH 6.6–8.2) [15, 42, 43].

### Kinetic parameters of wild-type and mutant *Da*CHI1

To investigate the substrate preference of *Da*CHI1, an enzymatic activity assay was performed in the presence of varying concentrations of two substrates, namely naringenin chalcone and isoliquiritigenin. The kinetic parameters of *Da*CHI1 are summarized in Table 4. Although wild-type *Da*CHI1 showed a strong preference for naringenin chalcone, the enzyme also catalyzed the isomerization of isoliquiritigenin, a type II CHI substrate. The *K*_m_ and *k*_cat_ values for isoliquiritigenin were 15.3 ± 1.5 μM and 1.3 ± 0.10 min^−1^, respectively, at 25°C, corresponding to a catalytic efficiency (*k*_cat_/*K*_m_) of 1416 M^−1^ s^−1^. The *K*_m_ value was comparable to those of type II CHIs from *M*. *sativa* and *Glycine max* for the same substrate (8.4 and 10 μM, respectively) [8, 27]. However, the *k*_cat_ *Da*CHI1 was considerably lower than those of *Ms*CHI and *Gm*CHI (8.1 × 10^6^ and 1.1 × 10^4^ min^−1^, respectively).

We also investigated the effects of mutating some important catalytic residues (R34, K108, and Y116) on enzymatic activity. R34A and R34M did not affect activity towards naringenin chalcone and only slightly affected the activity towards isoliquiritigenin. Compared to the wild type, K108A and Y116A showed *k*_cat_ values that were 2.3- and 13.5-fold lower against naringenin chalcone and 6.5- and 3.3-fold lower against isoliquiritigenin. The conserved hydrophobic residue Met36 interacted with isoliquiritigenin in the substrate-binding cleft of *Da*CHI1; mutating this residue to alanine lowered the catalytic efficiency for both substrates by ~2.8 fold, indicating that these residues are essential for the catalytic activity of *Da*CHI1.

We investigated the specific amino acid residues responsible for the difference in substrate preference between types I and II CHIs. In the α6 region of type II CHIs, two conserved residues—Thr190 and Met191—have been reported to affect substrate preference [22]. In type I CHIs, these residues are replaced with Ser and Ile, respectively. We therefore examined the role of Ser189 and Ile190 in the substrate preference of *Da*CHI1. Alanine substitution at Ser189 decreased *k*_cat_ against naringenin chalcone and isoliquiritigenin by 26 and 6.5 fold, respectively, relative to the wild type. When Ser189 and I190 were mutated to Thr and Met, respectively, which are present at the corresponding positions in type II CHIs, both mutants showed improved activity against naringenin chalcone (> 1.5 fold in *k*_cat_) relative to the wild type. However, when isoliquirigenin was used as the substrate, S189T showed comparable activity to the wild type whereas I190M showed a 4.3-fold decrease in *k*_cat_. Contrary to our expectation, the Ser189T and Ile190M mutations did not significantly affect the substrate preference of *Da*CHI1.

The sequence alignment revealed two conserved residues that differed between type I and II CHIs. In the latter, Lys and a Glu replaced Met96 and Gln104, respectively, of *Da*CHI1 and other type I CHIs. To determine whether these residues contribute to substrate preference, we generated M96K and Q104E mutants. The M96K mutation significantly reduced *k*_cat_ whereas Q104E showed comparable activity to the wild type towards both naringenin chalcone and isoliquiritigenin. None of the tested mutants significantly altered *K*_m_. Taken together, these results suggest that a single amino acid difference among CHI family members is not sufficient to determine substrate preference or classify the enzyme type.

## Conclusion

In this study, we showed that *Da*CHI1 expression is induced in response to environmental abiotic stressors such as low temperature or UV-B radiation, suggesting that *Da*CHI1 may play a role in the adaptation of plants to the polar environments. Furthermore, our analysis of enzymatic activity and structural data revealed how *Da*CHI1 accommodates and utilizes various substrates. This work revealed the novel substrate-binding mode of a type I CHI complexed with a type II substrate (isoliquiritigenin), clarifying the substrate specificity of CHI proteins. Furthermore, we provide structural evidence for the movement of a ligand from initial binding to deep binding within the CHI active site. We suggest that *Da*CHI1 may have evolved to use various substrates to produce sufficient flavonoids for a more rapid response to abiotic oxidative stresses. Our findings advance our understanding of how plants can survive in an extreme environment.

## Supporting information

S1 Fig DaCHI1 is monomer when analyzed in analytical ultracentrifugation (AUC). (PDF)

S1 File Validation report for PDB code 5YX3 and 5YX4. (PDF)
