# Supplementary material for: Crystal structure and enzymatic properties of chalcone isomerase from the Antarctic vascular plant Deschampsia antarctica Desv
Source: PLoS One. 2018 Feb 2;13(2):e0192415. doi: 10.1371/journal.pone.0192415 (PMC5796730; doi:10.1371/journal.pone.0192415)
Supplement: S1 Fig — (PDF) [file pone.0192415.s001.pdf]

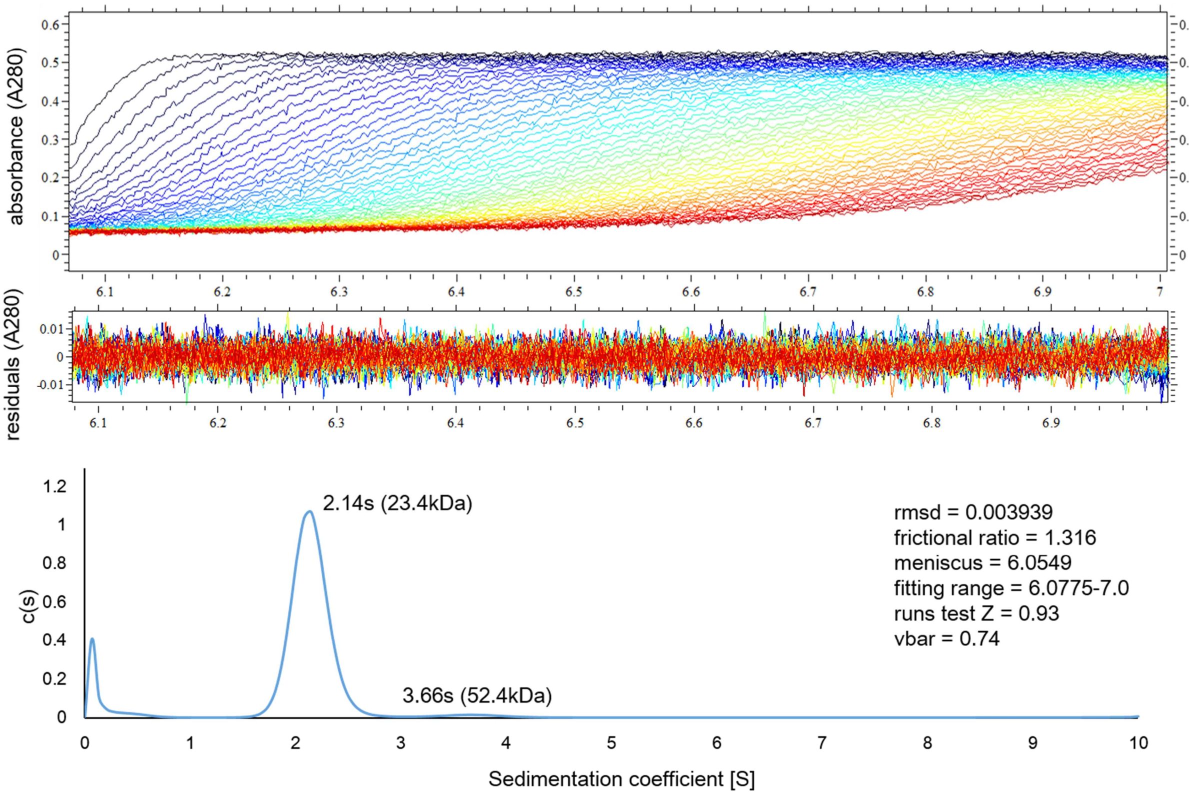

S1 Fig. Analytical ultracentrifugation (AUC) of *Da*CHI. Sedimentation velocity analysis of *Da*CHI was carried out with a XL-A analytical ultracentrifuge (Beckman Coulter, Brea, CA, USA). The protein was dissolved in a buffer of 20 mM Tris-HCl (pH 8.0), 150 mM NaCl. The sample and reference sectors of the dual-sector epon centerpiece were filled with the *Da*CHI protein solution and the buffer, respectively, and the cell was centrifuged (45,000 rpm). The sedimentation profile was monitored at 280 nm, and the experimental data were analyzed using the SEDFIT program [1,2]. *Da*CHI adopted a monomeric state in solution. AUC experiments using 0.5 mg/mL *Da*CHI (residues 1–231; calculated molecular weight of 23.9 kDa for the polypeptide chain) gives a mass of 23.4 kDa (sedimentation coefficient of 2.14 S and a frictional ratio of 1.316), indicating that *Da*CHI is a stable monomer in solution.

1. Schuck P. Size-distribution analysis of macromolecules by sedimentation velocity ultracentrifugation and lamm equation modeling. Biophys J. 2000; 78: 1606–1619.
2. Schuck P, Rossmanith P. Determination of the sedimentation coefficient distribution by least-squares boundary modeling. Biopolymers. 2000; 54: 328–341.
