# Supplementary material for: Crystal structure and enzymatic properties of chalcone isomerase from the Antarctic vascular plant Deschampsia antarctica Desv
Source: PLoS One. 2018 Feb 2;13(2):e0192415. doi: 10.1371/journal.pone.0192415 (PMC5796730; doi:10.1371/journal.pone.0192415)
Supplement: S1 File — (PDF) [file pone.0192415.s002.pdf]

Preliminary Full wwPDB X-ray Structure Validation Report (i)

Jan 15, 2018 – 04:10 AM EST

This is a Preliminary Full wwPDB X-ray Structure Validation Report.

This report is produced by the standalone wwPDB validation server.
The structure in question has not been deposited to the wwPDB.
This report should not be submitted to journals.

We welcome your comments at *validation@mail.wwpdb.org*
A user guide is available at
http://wwpdb.org/validation/2016/XrayValidationReportHelp
with specific help available everywhere you see the (i) symbol.

PRELIMINARY VALIDATION REPORT

The following versions of software and data (see references (i)) were used in the production of this report:

MolProbity	:	4.02b-467
Xtriage (Phenix)	:	1.9-1692
EDS	:	rb-20030736
Percentile statistics	:	20161228.v01 (using entries in the PDB archive December 28th 2016)
Refmac	:	5.8.0135
CCP4	:	6.5.0
Ideal geometry (proteins)	:	Engh & Huber (2001)
Ideal geometry (DNA, RNA)	:	Parkinson et al. (1996)
Validation Pipeline (wwPDB-VP)	:	rb-20030736

1 Overall quality at a glance (i)

The following experimental techniques were used to determine the structure:
X-RAY DIFFRACTION

The reported resolution of this entry is 2.70 Å.

Percentile scores (ranging between 0-100) for global validation metrics of the entry are shown in the following graphic. The table shows the number of entries on which the scores are based.

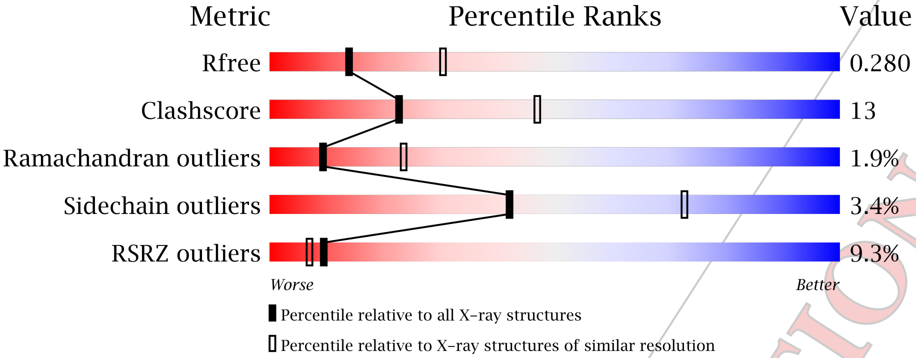

Metric	Whole archive (#Entries)	Similar resolution (#Entries, resolution range(Å))
R_{free}	100719	2259 (2.70-2.70)
Clashscore	112137	2590 (2.70-2.70)
Ramachandran outliers	110173	2550 (2.70-2.70)
Sidechain outliers	110143	2550 (2.70-2.70)
RSRZ outliers	101464	2275 (2.70-2.70)

The table below summarises the geometric issues observed across the polymeric chains and their fit to the electron density. The red, orange, yellow and green segments on the lower bar indicate the fraction of residues that contain outliers for >=3, 2, 1 and 0 types of geometric quality criteria. A grey segment represents the fraction of residues that are not modelled. The numeric value for each fraction is indicated below the corresponding segment, with a dot representing fractions <=5% The upper red bar (where present) indicates the fraction of residues that have poor fit to the electron density. The numeric value is given above the bar.

Mol	Chain	Length	Quality of chain
1	A	214	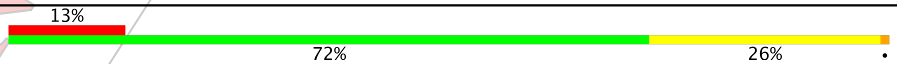
1	B	214	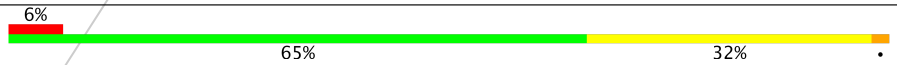

PRELIMINARY VALIDATION REPORT

2 Entry composition (i)

There are 2 unique types of molecules in this entry. The entry contains 3155 atoms, of which 0 are hydrogens and 0 are deuteriums.

In the tables below, the ZeroOcc column contains the number of atoms modelled with zero occupancy, the AltConf column contains the number of residues with at least one atom in alternate conformation and the Trace column contains the number of residues modelled with at most 2 atoms.

- Molecule 1 is a protein.

Mol	Chain	Residues	Atoms					ZeroOcc	AltConf	Trace
1	A	214	Total<br>1577	C<br>1010	N<br>259	O<br>304	S<br>4	0	0	0
1	B	214	Total<br>1577	C<br>1010	N<br>259	O<br>304	S<br>4	0	0	0

- Molecule 2 is water.

Mol	Chain	Residues	Atoms		ZeroOcc	AltConf
2	S	1	Total<br>1	O<br>1	0	0

PRELIMINARY VALIDATION REPORT

3 Residue-property plots (i)

These plots are drawn for all protein, RNA and DNA chains in the entry. The first graphic for a chain summarises the proportions of the various outlier classes displayed in the second graphic. The second graphic shows the sequence view annotated by issues in geometry and electron density. Residues are color-coded according to the number of geometric quality criteria for which they contain at least one outlier: green = 0, yellow = 1, orange = 2 and red = 3 or more. A red dot above a residue indicates a poor fit to the electron density (RSRZ > 2). Stretches of 2 or more consecutive residues without any outlier are shown as a green connector. Residues present in the sample, but not in the model, are shown in grey.

- Molecule 1:

Chain A:

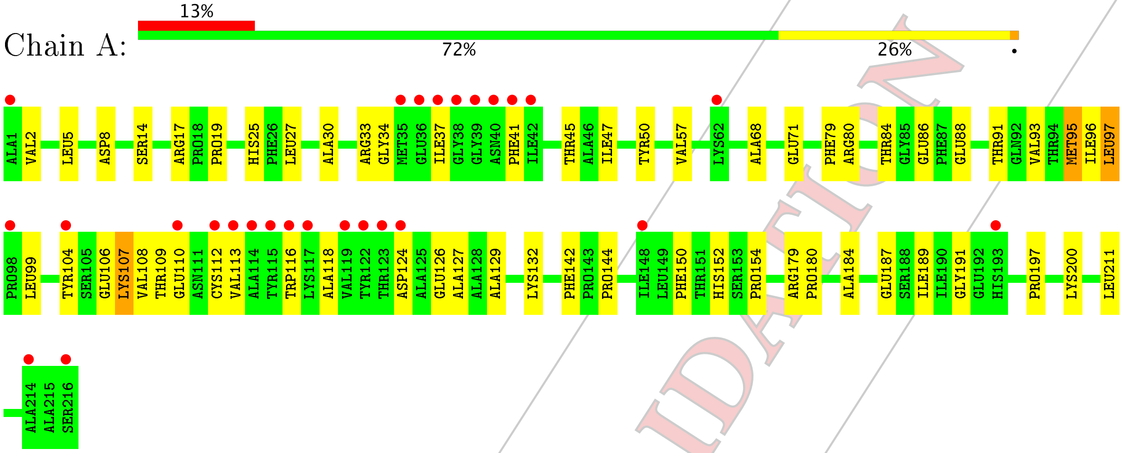

- Molecule 1:

Chain B:

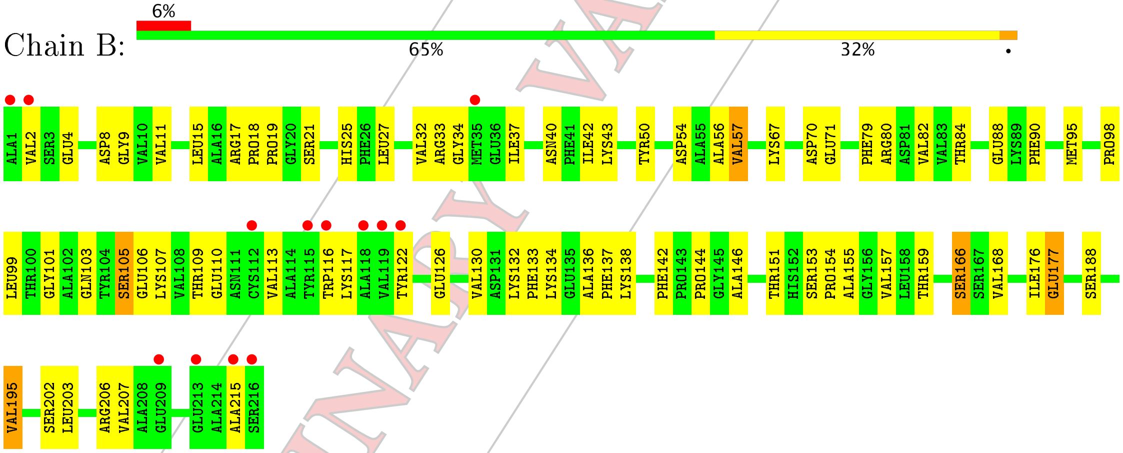

PRELIMINARY VALIDATION REPORT

4 Data and refinement statistics ⓘ

Property	Value	Source
Space group	C 2 2 21	Depositor
Cell constants<br>a, b, c, α, β, γ	103.47Å 113.64Å 76.72Å<br>90.00° 90.00° 90.00°	Depositor
Resolution (Å)	32.27 – 2.70<br>32.27 – 2.70	Depositor<br>EDS
% Data completeness<br>(in resolution range)	97.8 (32.27-2.70)<br>97.8 (32.27-2.70)	Depositor<br>EDS
R_{merge}	(Not available)	Depositor
R_{sym}	(Not available)	Depositor
$< I/\sigma(I) >$ [1]	11.02 (at 2.68Å)	Xtriage
Refinement program	PHENIX 1.9_1692	Depositor
R, R_{free}	0.222 , 0.278<br>0.215 , 0.280	Depositor<br>DCC
R_{free} test set	607 reflections (4.86%)	DCC
Wilson B-factor (Å^2)	56.6	Xtriage
Anisotropy	0.976	Xtriage
Bulk solvent k_{sol}(e/Å^3), B_{sol}(Å^2)	0.34 , 61.7	EDS
L-test for twinning[2]	$< \|L\| > = 0.51$, $< L^2 > = 0.35$	Xtriage
Estimated twinning fraction	No twinning to report.	Xtriage
F_o,F_c correlation	0.94	EDS
Total number of atoms	3155	wwPDB-VP
Average B, all atoms (Å^2)	77.0	wwPDB-VP

Xtriage's analysis on translational NCS is as follows: *The largest off-origin peak in the Patterson function is 5.08% of the height of the origin peak. No significant pseudotranslation is detected.*

[1] Intensities estimated from amplitudes.

[2] Theoretical values of $< |L| >$, $< L^2 >$ for acentric reflections are 0.5, 0.333 respectively for untwinned datasets, and 0.375, 0.2 for perfectly twinned datasets.

PRELIMINARY VALIDATION REPORT

5 Model quality (i)

5.1 Standard geometry (i)

The Z score for a bond length (or angle) is the number of standard deviations the observed value is removed from the expected value. A bond length (or angle) with $|Z| > 5$ is considered an outlier worth inspection. RMSZ is the root-mean-square of all Z scores of the bond lengths (or angles).

Mol	Chain	Bond lengths		Bond angles	
		RMSZ	#$|Z|$ >5	RMSZ	#$|Z|$ >5
1	A	0.39	0/1610	0.61	0/2186
1	B	0.31	0/1610	0.54	0/2186
All	All	0.35	0/3220	0.58	0/4372

There are no bond length outliers.

There are no bond angle outliers.

There are no chirality outliers.

There are no planarity outliers.

5.2 Too-close contacts (i)

In the following table, the Non-H and H(model) columns list the number of non-hydrogen atoms and hydrogen atoms in the chain respectively. The H(added) column lists the number of hydrogen atoms added and optimized by MolProbity. The Clashes column lists the number of clashes within the asymmetric unit, whereas Symm-Clashes lists symmetry related clashes.

Mol	Chain	Non-H	H(model)	H(added)	Clashes	Symm-Clashes
1	A	1577	0	1567	40	0
1	B	1577	0	1567	44	0
2	S	1	0	0	1	0
All	All	3155	0	3134	82	0

The all-atom clashscore is defined as the number of clashes found per 1000 atoms (including hydrogen atoms). The all-atom clashscore for this structure is 13.

All (82) close contacts within the same asymmetric unit are listed below, sorted by their clash magnitude.

Atom-1	Atom-2	Interatomic distance (Å)	Clash overlap (Å)
1:B:33:ARG:HD3	1:B:195:VAL:HG13	1.55	0.88

Continued on next page...

PRELIMINARY VALIDATION REPORT

Continued from previous page...

Atom-1	Atom-2	Interatomic distance (Å)	Clash overlap (Å)
1:A:107:LYS:HE3	1:A:108:VAL:HB	1.60	0.82
1:A:107:LYS:HG2	1:A:108:VAL:N	1.98	0.75
1:B:33:ARG:NH1	1:B:34:GLY:O	2.19	0.75
1:A:2:VAL:HG23	1:A:14:SER:HB2	1.68	0.74
1:A:5:LEU:HB2	1:A:27:LEU:HD21	1.67	0.74
1:A:88:GLU:HB3	1:A:154:PRO:HD3	1.70	0.73
1:A:68:ALA:N	1:A:71:GLU:OE1	2.22	0.69
1:A:197:PRO:HA	1:A:200:LYS:HD2	1.73	0.69
1:B:25:HIS:HB3	1:B:50:TYR:HB3	1.80	0.64
1:B:134:LYS:O	1:B:138:LYS:HG3	1.98	0.64
1:B:106:GLU:O	1:B:110:GLU:HG2	1.98	0.63
1:A:93:VAL:HG21	1:A:104:TYR:OH	1.99	0.62
1:A:5:LEU:HD23	1:A:30:ALA:HB3	1.84	0.59
1:A:33:ARG:HB3	1:A:45:THR:HB	1.85	0.59
1:B:116:TRP:HB3	1:B:122:TYR:HB2	1.84	0.59
1:B:101:GLY:O	1:B:105:SER:OG	2.16	0.58
1:B:113:VAL:O	1:B:117:LYS:HG2	2.04	0.58
1:A:187:GLU:HG3	1:A:191:GLY:HA3	1.85	0.57
1:B:88:GLU:HG3	1:B:153:SER:HA	1.87	0.55
1:A:104:TYR:CE2	1:A:107:LYS:HE2	2.42	0.55
1:A:86:GLU:HG3	1:A:86:GLU:O	2.05	0.55
1:A:126:GLU:OE1	1:A:126:GLU:N	2.40	0.54
1:B:134:LYS:HB2	1:B:138:LYS:HE2	1.89	0.54
1:B:151:THR:HB	1:B:159:THR:HB	1.89	0.54
1:B:99:LEU:HG	1:B:103:GLN:HE21	1.73	0.54
1:B:202:SER:O	1:B:206:ARG:HG3	2.07	0.54
1:A:34:GLY:HA3	1:A:41:PHE:CZ	2.45	0.51
1:B:106:GLU:HG2	1:B:107:LYS:HG3	1.92	0.51
1:B:33:ARG:CD	1:B:195:VAL:HG13	2.36	0.51
1:B:37:ILE:HB	1:B:42:ILE:HD11	1.91	0.51
1:A:96:ILE:O	1:A:97:LEU:HB2	2.11	0.51
1:B:56:ALA:O	1:B:57:VAL:HB	2.12	0.50
1:B:80:ARG:O	1:B:84:THR:OG1	2.24	0.50
1:A:179:ARG:HB3	1:A:180:PRO:HD3	1.93	0.49
1:A:57:VAL:HG13	1:A:211:LEU:HD23	1.94	0.49
1:B:32:VAL:HG21	1:B:43:LYS:HG2	1.94	0.49
1:B:98:PRO:HG3	1:B:144:PRO:HD3	1.95	0.49
1:B:188:SER:O	1:B:195:VAL:HB	2.13	0.48
1:B:79:PHE:HA	1:B:82:VAL:HG12	1.95	0.48
1:A:113:VAL:HA	1:A:116:TRP:HB2	1.96	0.48
1:A:110:GLU:O	1:A:113:VAL:N	2.47	0.48

Continued on next page...

PRELIMINARY VALIDATION REPORT

Continued from previous page...

Atom-1	Atom-2	Interatomic distance (Å)	Clash overlap (Å)
1:B:18:PRO:HB3	1:B:90:PHE:CD1	2.49	0.47
1:A:104:TYR:CD2	1:A:107:LYS:HE2	2.49	0.47
1:A:47:ILE:HG21	1:A:189:ILE:HG21	1.96	0.47
1:A:91:THR:HG23	1:A:150:PHE:HB2	1.95	0.47
1:A:91:THR:HG22	1:A:152:HIS:NE2	2.30	0.47
1:A:109:THR:O	1:A:113:VAL:HG22	2.14	0.47
1:B:40:ASN:ND2	2:S:1:HOH:O	2.39	0.46
1:A:110:GLU:HA	1:A:113:VAL:CG2	2.45	0.46
1:B:157:VAL:HG22	1:B:177:GLU:HB3	1.97	0.46
1:A:25:HIS:HB3	1:A:50:TYR:HB3	1.96	0.46
1:B:132:LYS:HE3	1:B:176:ILE:HG12	1.98	0.46
1:A:124:ASP:O	1:A:127:ALA:N	2.50	0.45
1:B:27:LEU:HG	1:B:206:ARG:NH1	2.30	0.45
1:B:95:MET:HG3	1:B:142:PHE:HB3	1.97	0.45
1:B:95:MET:HG2	1:B:146:ALA:O	2.16	0.45
1:A:107:LYS:CE	1:A:108:VAL:HB	2.39	0.45
1:B:166:SER:O	1:B:166:SER:OG	2.35	0.44
1:B:88:GLU:HG3	1:B:154:PRO:HD3	1.98	0.44
1:A:129:ALA:HA	1:A:132:LYS:HE2	1.99	0.44
1:B:109:THR:HG21	1:B:134:LYS:NZ	2.33	0.44
1:B:101:GLY:HA3	1:B:137:PHE:O	2.18	0.44
1:B:21:SER:OG	1:B:88:GLU:OE1	2.17	0.43
1:A:104:TYR:O	1:A:107:LYS:N	2.51	0.43
1:A:80:ARG:O	1:A:84:THR:HB	2.18	0.43
1:A:79:PHE:CZ	1:A:200:LYS:HB3	2.54	0.43
1:B:133:PHE:O	1:B:136:ALA:HB3	2.19	0.43
1:B:126:GLU:N	1:B:126:GLU:OE1	2.51	0.43
1:B:70:ASP:OD1	1:B:70:ASP:N	2.52	0.42
1:B:15:LEU:HD23	1:B:25:HIS:O	2.18	0.42
1:A:17:ARG:O	1:B:19:PRO:HB3	2.20	0.42
1:A:45:THR:HG21	1:A:93:VAL:HG13	2.02	0.41
1:B:67:LYS:HG2	1:B:71:GLU:HB3	2.02	0.41
1:A:19:PRO:HB3	1:B:17:ARG:O	2.20	0.41
1:B:8:ASP:OD2	1:B:9:GLY:N	2.51	0.41
1:A:96:ILE:H	1:A:144:PRO:HA	1.85	0.41
1:A:112:CYS:SG	1:A:184:ALA:HB1	2.61	0.41
1:A:95:MET:HG3	1:A:142:PHE:HB3	2.03	0.40
1:A:37:ILE:HD13	1:A:37:ILE:HA	1.97	0.40
1:B:130:VAL:O	1:B:134:LYS:HG2	2.22	0.40
1:B:203:LEU:O	1:B:207:VAL:HG23	2.22	0.40

There are no symmetry-related clashes.

PRELIMINARY VALIDATION REPORT

PRELIMINARY VALIDATION REPORT

5.3 Torsion angles (i)

5.3.1 Protein backbone (i)

In the following table, the Percentiles column shows the percent Ramachandran outliers of the chain as a percentile score with respect to all X-ray entries followed by that with respect to entries of similar resolution.

The Analysed column shows the number of residues for which the backbone conformation was analysed, and the total number of residues.

Mol	Chain	Analysed	Favoured	Allowed	Outliers	Percentiles	
1	A	210/214 (98%)	192 (91%)	14 (7%)	4 (2%)	9	23
1	B	210/214 (98%)	198 (94%)	8 (4%)	4 (2%)	9	23
All	All	420/428 (98%)	390 (93%)	22 (5%)	8 (2%)	9	23

All (8) Ramachandran outliers are listed below:

Mol	Chain	Res	Type
1	A	97	LEU
1	B	155	ALA
1	A	95	MET
1	B	2	VAL
1	B	215	ALA
1	A	8	ASP
1	A	118	ALA
1	B	57	VAL

5.3.2 Protein sidechains (i)

In the following table, the Percentiles column shows the percent sidechain outliers of the chain as a percentile score with respect to all X-ray entries followed by that with respect to entries of similar resolution.

The Analysed column shows the number of residues for which the sidechain conformation was analysed, and the total number of residues.

Mol	Chain	Analysed	Rotameric	Outliers	Percentiles	
1	A	160/160 (100%)	157 (98%)	3 (2%)	62	87
1	B	160/160 (100%)	152 (95%)	8 (5%)	28	57
All	All	320/320 (100%)	309 (97%)	11 (3%)	42	73

All (11) residues with a non-rotameric sidechain are listed below:

Mol	Chain	Res	Type
1	A	99	LEU
1	A	106	GLU
1	A	107	LYS
1	B	4	GLU
1	B	11	VAL
1	B	54	ASP
1	B	105	SER
1	B	166	SER
1	B	168	VAL
1	B	177	GLU
1	B	195	VAL

Some sidechains can be flipped to improve hydrogen bonding and reduce clashes. There are no such sidechains identified.

5.3.3 RNA (i)

There are no RNA molecules in this entry.

5.4 Non-standard residues in protein, DNA, RNA chains (i)

There are no non-standard protein/DNA/RNA residues in this entry.

5.5 Carbohydrates (i)

There are no carbohydrates in this entry.

5.6 Ligand geometry (i)

There are no ligands in this entry.

5.7 Other polymers (i)

There are no such residues in this entry.

5.8 Polymer linkage issues (i)

The following chains have linkage breaks:

PRELIMINARY VALIDATION REPORT

Mol	Chain	Number of breaks
1	B	1
1	A	1

All chain breaks are listed below:

Model	Chain	Residue-1	Atom-1	Residue-2	Atom-2	Distance (Å)
1	B	119:VAL	C	122:TYR	N	5.50
1	A	119:VAL	C	122:TYR	N	5.27

PRELIMINARY VALIDATION REPORT

6 Fit of model and data (i)

6.1 Protein, DNA and RNA chains (i)

In the following table, the column labelled '#RSRZ> 2' contains the number (and percentage) of RSRZ outliers, followed by percent RSRZ outliers for the chain as percentile scores relative to all X-ray entries and entries of similar resolution. The OWAB column contains the minimum, median, 95th percentile and maximum values of the occupancy-weighted average B-factor per residue. The column labelled 'Q< 0.9' lists the number of (and percentage) of residues with an average occupancy less than 0.9.

Mol	Chain	Analysed	<RSRZ>	#RSRZ>2			OWAB(Å^2)	Q<0.9
1	A	214/214 (100%)	0.86	27 (12%)	4	3	51, 73, 119, 133	0
1	B	214/214 (100%)	0.47	13 (6%)	22	20	53, 71, 109, 120	0
All	All	428/428 (100%)	0.66	40 (9%)	9	7	51, 71, 113, 133	0

All (40) RSRZ outliers are listed below:

Mol	Chain	Res	Type	RSRZ
1	A	119	VAL	5.4
1	A	116	TRP	5.3
1	A	115	TYR	4.8
1	A	42	ILE	4.7
1	A	36	GLU	4.7
1	A	112	CYS	4.3
1	A	113	VAL	4.2
1	B	115	TYR	3.9
1	A	39	GLY	3.9
1	A	110	GLU	3.8
1	A	38	GLY	3.7
1	B	215	ALA	3.6
1	A	37	ILE	3.6
1	A	122	TYR	3.6
1	A	41	PHE	3.5
1	B	118	ALA	3.4
1	B	2	VAL	3.3
1	A	123	THR	3.3
1	A	1	ALA	3.3
1	A	214	ALA	3.2
1	B	119	VAL	3.1
1	B	216	SER	3.1
1	A	114	ALA	3.0
1	A	35	MET	2.8

Continued on next page...

PRELIMINARY VALIDATION REPORT

Continued from previous page...

Mol	Chain	Res	Type	RSRZ
1	A	117	LYS	2.6
1	B	112	CYS	2.6
1	A	104	TYR	2.5
1	B	1	ALA	2.4
1	B	209	GLU	2.4
1	A	62	LYS	2.4
1	B	213	GLU	2.4
1	A	193	HIS	2.2
1	A	40	ASN	2.2
1	B	122	TYR	2.2
1	B	35	MET	2.2
1	A	124	ASP	2.1
1	A	98	PRO	2.1
1	A	148	ILE	2.1
1	B	116	TRP	2.1
1	A	216	SER	2.0

6.2 Non-standard residues in protein, DNA, RNA chains (i)

There are no non-standard protein/DNA/RNA residues in this entry.

6.3 Carbohydrates (i)

There are no carbohydrates in this entry.

6.4 Ligands (i)

There are no ligands in this entry.

6.5 Other polymers (i)

There are no such residues in this entry.

PRELIMINARY VALIDATION REPORT

Preliminary Full wwPDB X-ray Structure Validation Report (i)

Dec 1, 2017 – 02:07 PM JST

Deposition ID	:	D_1300006058
PDB ID	:	*(not yet assigned)*

This is a Preliminary Full wwPDB X-ray Structure Validation Report.

This report is produced by the wwPDB Deposition System during initial deposition but before annotation of the structure.

We welcome your comments at *validation@mail.wwpdb.org*
A user guide is available at
http://wwpdb.org/validation/2016/XrayValidationReportHelp
with specific help available everywhere you see the (i) symbol.

The following versions of software and data (see references (i)) were used in the production of this report:

MolProbity	:	4.02b-467
Mogul	:	1.7.2 (RC1), CSD as538be (2017)
Xtriage (Phenix)	:	1.9-1692
EDS	:	rb-20030345
Percentile statistics	:	20161228.v01 (using entries in the PDB archive December 28th 2016)
Refmac	:	5.8.0135
CCP4	:	6.5.0
Ideal geometry (proteins)	:	Engh & Huber (2001)
Ideal geometry (DNA, RNA)	:	Parkinson et al. (1996)
Validation Pipeline (wwPDB-VP)	:	rb-20030345

1 Overall quality at a glance (i)

The following experimental techniques were used to determine the structure:
X-RAY DIFFRACTION

The reported resolution of this entry is 2.10 Å.

Percentile scores (ranging between 0-100) for global validation metrics of the entry are shown in the following graphic. The table shows the number of entries on which the scores are based.

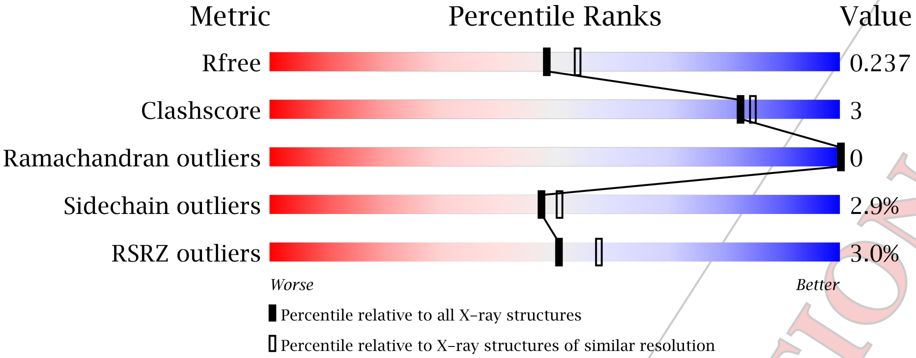

Metric	Whole archive (#Entries)	Similar resolution (#Entries, resolution range(Å))
R_{free}	100719	4243 (2.10-2.10)
Clashscore	112137	4788 (2.10-2.10)
Ramachandran outliers	110173	4740 (2.10-2.10)
Sidechain outliers	110143	4741 (2.10-2.10)
RSRZ outliers	101464	4275 (2.10-2.10)

The table below summarises the geometric issues observed across the polymeric chains and their fit to the electron density. The red, orange, yellow and green segments on the lower bar indicate the fraction of residues that contain outliers for >=3, 2, 1 and 0 types of geometric quality criteria. A grey segment represents the fraction of residues that are not modelled. The numeric value for each fraction is indicated below the corresponding segment, with a dot representing fractions <=5% The upper red bar (where present) indicates the fraction of residues that have poor fit to the electron density. The numeric value is given above the bar.

Mol	Chain	Length	Quality of chain
1	A	232	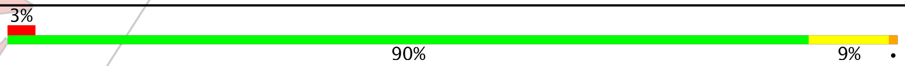

PRELIMINARY VALIDATION REPORT

2 Entry composition (i)

There are 3 unique types of molecules in this entry. The entry contains 1931 atoms, of which 0 are hydrogens and 0 are deuteriums.

In the tables below, the ZeroOcc column contains the number of atoms modelled with zero occupancy, the AltConf column contains the number of residues with at least one atom in alternate conformation and the Trace column contains the number of residues modelled with at most 2 atoms.

- Molecule 1 is a protein.

Mol	Chain	Residues	Atoms					ZeroOcc	AltConf	Trace
			Total	C	N	O	S			
1	A	232	1694	1082	277	329	6	0	0	0

- Molecule 2 is 2',4,4'-TRIHYDROXYCHALCONE (three-letter code: HCC) (formula: $C_{15}H_{12}O_4$).

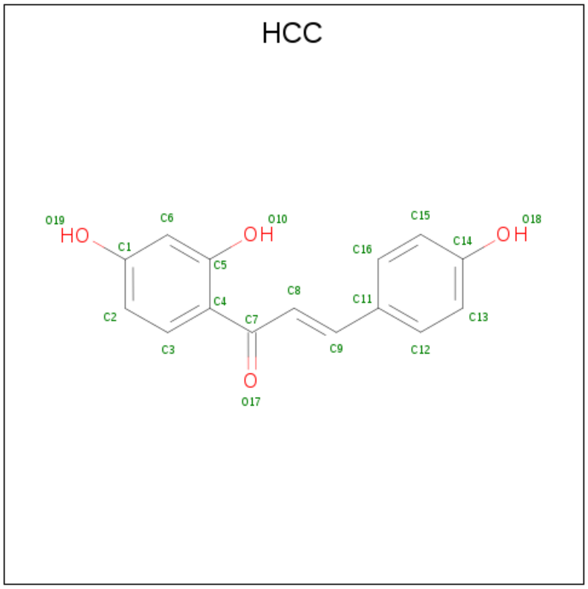

Mol	Chain	Residues	Atoms			ZeroOcc	AltConf
			Total	C	O		
2	B	1	19	15	4	0	0

- Molecule 3 is water.

Mol	Chain	Residues	Atoms		ZeroOcc	AltConf
			Total	O		
3	S	218	218	218	0	0

PRELIMINARY VALIDATION REPORT

3 Residue-property plots (i)

These plots are drawn for all protein, RNA and DNA chains in the entry. The first graphic for a chain summarises the proportions of the various outlier classes displayed in the second graphic. The second graphic shows the sequence view annotated by issues in geometry and electron density. Residues are color-coded according to the number of geometric quality criteria for which they contain at least one outlier: green = 0, yellow = 1, orange = 2 and red = 3 or more. A red dot above a residue indicates a poor fit to the electron density (RSRZ > 2). Stretches of 2 or more consecutive residues without any outlier are shown as a green connector. Residues present in the sample, but not in the model, are shown in grey.

- Molecule 1:

Chain A:

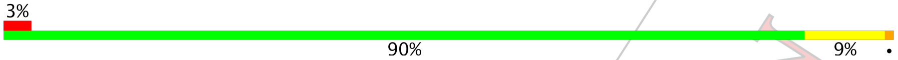

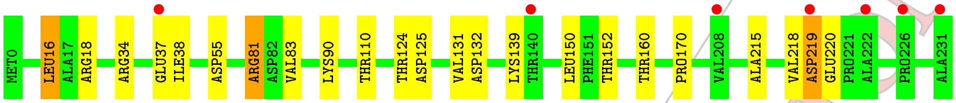

PRELIMINARY VALIDATION REPORT

4 Data and refinement statistics (i)

Property	Value	Source
Space group	P 32 2 1	Depositor
Cell constants<br>a, b, c, α, β, γ	83.22Å 83.22Å 93.13Å<br>90.00° 90.00° 120.00°	Depositor
Resolution (Å)	41.61 – 2.10<br>39.11 – 2.10	Depositor<br>EDS
% Data completeness<br>(in resolution range)	99.4 (41.61-2.10)<br>99.5 (39.11-2.10)	Depositor<br>EDS
R_{merge}	(Not available)	Depositor
R_{sym}	(Not available)	Depositor
$< I/\sigma(I) >$ [1]	3.13 (at 2.10Å)	Xtriage
Refinement program	REFMAC 5.8.0158	Depositor
R, R_{free}	0.189 , 0.226<br>0.200 , 0.237	Depositor<br>DCC
R_{free} test set	1154 reflections (5.50%)	DCC
Wilson B-factor (Å^2)	48.2	Xtriage
Anisotropy	0.024	Xtriage
Bulk solvent k_{sol}(e/Å^3), B_{sol}(Å^2)	0.36 , 51.7	EDS
L-test for twinning[2]	$< \lvert L \rvert > = 0.49$, $< L^2 > = 0.32$	Xtriage
Estimated twinning fraction	0.029 for -h,-k,l	Xtriage
F_o,F_c correlation	0.95	EDS
Total number of atoms	1931	wwPDB-VP
Average B, all atoms (Å^2)	55.0	wwPDB-VP

Xtriage's analysis on translational NCS is as follows: *The largest off-origin peak in the Patterson function is 6.42% of the height of the origin peak. No significant pseudotranslation is detected.*

[1] Intensities estimated from amplitudes.
[2] Theoretical values of $< \lvert L \rvert >$, $< L^2 >$ for acentric reflections are 0.5, 0.333 respectively for untwinned datasets, and 0.375, 0.2 for perfectly twinned datasets.

PRELIMINARY VALIDATION REPORT

5 Model quality (i)

5.1 Standard geometry (i)

Bond lengths and bond angles in the following residue types are not validated in this section: HCC

The Z score for a bond length (or angle) is the number of standard deviations the observed value is removed from the expected value. A bond length (or angle) with $|Z| > 5$ is considered an outlier worth inspection. RMSZ is the root-mean-square of all Z scores of the bond lengths (or angles).

Mol	Chain	Bond lengths		Bond angles	
		RMSZ	#$\|Z\|$ >5	RMSZ	#$\|Z\|$ >5
1	A	0.99	0/1730	0.96	5/2353 (0.2%)

Chiral center outliers are detected by calculating the chiral volume of a chiral center and verifying if the center is modelled as a planar moiety or with the opposite hand.A planarity outlier is detected by checking planarity of atoms in a peptide group, atoms in a mainchain group or atoms of a sidechain that are expected to be planar.

Mol	Chain	#Chirality outliers	#Planarity outliers
1	A	0	1

There are no bond length outliers.

All (5) bond angle outliers are listed below:

Mol	Chain	Res	Type	Atoms	Z	Observed(^{o})	Ideal(^{o})
1	A	81	ARG	NE-CZ-NH1	7.07	123.83	120.30
1	A	18	ARG	NE-CZ-NH2	6.46	123.53	120.30
1	A	55	ASP	CB-CA-C	-6.19	98.01	110.40
1	A	81	ARG	NE-CZ-NH2	-5.95	117.32	120.30
1	A	16	LEU	CA-CB-CG	5.20	127.26	115.30

There are no chirality outliers.

All (1) planarity outliers are listed below:

Mol	Chain	Res	Type	Group
1	A	38	ILE	Peptide

PRELIMINARY VALIDATION REPORT

5.2 Too-close contacts (i)

In the following table, the Non-H and H(model) columns list the number of non-hydrogen atoms and hydrogen atoms in the chain respectively. The H(added) column lists the number of hydrogen atoms added and optimized by MolProbity. The Clashes column lists the number of clashes within the asymmetric unit, whereas Symm-Clashes lists symmetry related clashes.

Mol	Chain	Non-H	H(model)	H(added)	Clashes	Symm-Clashes
1	A	1694	0	1681	10	0
2	B	19	0	9	0	0
3	S	218	0	0	3	0
All	All	1931	0	1690	10	0

The all-atom clashscore is defined as the number of clashes found per 1000 atoms (including hydrogen atoms). The all-atom clashscore for this structure is 3.

All (10) close contacts within the same asymmetric unit are listed below, sorted by their clash magnitude.

Atom-1	Atom-2	Interatomic distance (Å)	Clash overlap (Å)
1:A:139:LYS:O	3:S:167:HOH:O	2.15	0.63
1:A:132:ASP:OD1	3:S:158:HOH:O	2.17	0.57
1:A:150:LEU:HD13	1:A:170:PRO:HD2	1.87	0.55
1:A:220:GLU:OE2	3:S:56:HOH:O	2.19	0.52
1:A:152:THR:HB	1:A:160:THR:HB	1.96	0.48
1:A:218:VAL:HG23	1:A:219:ASP:N	2.31	0.45
1:A:215:ALA:O	1:A:218:VAL:HG22	2.18	0.44
1:A:124:THR:HG22	1:A:125:ASP:N	2.34	0.42
1:A:83:VAL:O	1:A:90:LYS:HE2	2.21	0.40
1:A:110:THR:CG2	1:A:131:VAL:HG13	2.52	0.40

There are no symmetry-related clashes.

5.3 Torsion angles (i)

5.3.1 Protein backbone (i)

In the following table, the Percentiles column shows the percent Ramachandran outliers of the chain as a percentile score with respect to all X-ray entries followed by that with respect to entries of similar resolution.

The Analysed column shows the number of residues for which the backbone conformation was analysed, and the total number of residues.

PRELIMINARY VALIDATION REPORT

Mol	Chain	Analysed	Favoured	Allowed	Outliers	Percentiles	
1	A	230/232 (99%)	224 (97%)	6 (3%)	0	100	100

There are no Ramachandran outliers to report.

5.3.2 Protein sidechains (i)

In the following table, the Percentiles column shows the percent sidechain outliers of the chain as a percentile score with respect to all X-ray entries followed by that with respect to entries of similar resolution.

The Analysed column shows the number of residues for which the sidechain conformation was analysed, and the total number of residues.

Mol	Chain	Analysed	Rotameric	Outliers	Percentiles	
1	A	171/171 (100%)	166 (97%)	5 (3%)	48	51

All (5) residues with a non-rotameric sidechain are listed below:

Mol	Chain	Res	Type
1	A	16	LEU
1	A	34	ARG
1	A	37	GLU
1	A	81	ARG
1	A	219	ASP

Some sidechains can be flipped to improve hydrogen bonding and reduce clashes. There are no such sidechains identified.

5.3.3 RNA (i)

There are no RNA molecules in this entry.

5.4 Non-standard residues in protein, DNA, RNA chains (i)

There are no non-standard protein/DNA/RNA residues in this entry.

5.5 Carbohydrates (i)

There are no carbohydrates in this entry.

PRELIMINARY VALIDATION REPORT

5.6 Ligand geometry (i)

1 ligand is modelled in this entry.

In the following table, the Counts columns list the number of bonds (or angles) for which Mogul statistics could be retrieved, the number of bonds (or angles) that are observed in the model and the number of bonds (or angles) that are defined in the Chemical Component Dictionary. The Link column lists molecule types, if any, to which the group is linked. The Z score for a bond length (or angle) is the number of standard deviations the observed value is removed from the expected value. A bond length (or angle) with $|Z| > 2$ is considered an outlier worth inspection. RMSZ is the root-mean-square of all Z scores of the bond lengths (or angles).

Mol	Type	Chain	Res	Link	Bond lengths			Bond angles		
					Counts	RMSZ	$\#\|Z\| > 2$	Counts	RMSZ	$\#\|Z\| > 2$
2	HCC	B	1	-	20,20,20	1.69	2 (10%)	27,27,27	0.95	0

In the following table, the Chirals column lists the number of chiral outliers, the number of chiral centers analysed, the number of these observed in the model and the number defined in the Chemical Component Dictionary. Similar counts are reported in the Torsion and Rings columns. '-' means no outliers of that kind were identified.

Mol	Type	Chain	Res	Link	Chirals	Torsions	Rings
2	HCC	B	1	-	-	0/9/9/9	0/2/2/2

All (2) bond length outliers are listed below:

Mol	Chain	Res	Type	Atoms	Z	Observed(Å)	Ideal(Å)
2	B	1	HCC	C4-C7	2.13	1.52	1.48
2	B	1	HCC	C4-C5	6.50	1.51	1.41

There are no bond angle outliers.

There are no chirality outliers.

There are no torsion outliers.

There are no ring outliers.

No monomer is involved in short contacts.

5.7 Other polymers (i)

There are no such residues in this entry.

PRELIMINARY VALIDATION REPORT

5.8 Polymer linkage issues (i)

There are no chain breaks in this entry.

PRELIMINARY VALIDATION REPORT

6 Fit of model and data (i)

6.1 Protein, DNA and RNA chains (i)

In the following table, the column labelled '#RSRZ> 2' contains the number (and percentage) of RSRZ outliers, followed by percent RSRZ outliers for the chain as percentile scores relative to all X-ray entries and entries of similar resolution. The OWAB column contains the minimum, median, 95th percentile and maximum values of the occupancy-weighted average B-factor per residue. The column labelled 'Q< 0.9' lists the number of (and percentage) of residues with an average occupancy less than 0.9.

Mol	Chain	Analysed	<RSRZ>	#RSRZ>2	OWAB(Å^2)	Q<0.9
1	A	232/232 (100%)	-0.04	7 (3%) 51 58	39, 51, 78, 111	0

All (7) RSRZ outliers are listed below:

Mol	Chain	Res	Type	RSRZ
1	A	219	ASP	4.2
1	A	140	THR	3.5
1	A	231	ALA	3.3
1	A	226	PRO	2.7
1	A	208	VAL	2.5
1	A	37	GLU	2.3
1	A	222	ALA	2.1

6.2 Non-standard residues in protein, DNA, RNA chains (i)

There are no non-standard protein/DNA/RNA residues in this entry.

6.3 Carbohydrates (i)

There are no carbohydrates in this entry.

6.4 Ligands (i)

In the following table, the Atoms column lists the number of modelled atoms in the group and the number defined in the chemical component dictionary. LLDF column lists the quality of electron density of the group with respect to its neighbouring residues in protein, DNA or RNA chains. The B-factors column lists the minimum, median, 95th percentile and maximum values of B factors of atoms in the group. The column labelled 'Q< 0.9' lists the number of atoms with occupancy less than 0.9.

PRELIMINARY VALIDATION REPORT

Mol	Type	Chain	Res	Atoms	RSCC	RSR	LLDF	B-factors($Å^2$)	Q<0.9
2	HCC	B	1	19/?	0.95	0.08	-1.17	43,49,54,55	0

6.5 Other polymers ⓘ

There are no such residues in this entry.

PRELIMINARY VALIDATION REPORT
